# Homogeneity of antibody-drug conjugates critically impacts the therapeutic efficacy in brain tumors

**DOI:** 10.1016/j.celrep.2022.110839

**Published:** 2022-05-24

**Authors:** Yasuaki Anami, Yoshihiro Otani, Wei Xiong, Summer Y.Y. Ha, Aiko Yamaguchi, Kimberly A. Rivera-Caraballo, Ningyan Zhang, Zhiqiang An, Balveen Kaur, Kyoji Tsuchikama

**Affiliations:** 1 Texas Therapeutics Institute, The Brown Foundation Institute of Molecular Medicine, McGovern Medical School, The University of Texas Health Center at Houston, Houston, TX 77054, USA; 2 Department of Neurosurgery, McGovern Medical School, The University of Texas Health Science Center at Houston, Houston, TX 77030, USA; 3 Lead contact

## Abstract

Glioblastoma multiforme (GBM) is the most aggressive and fatal disease of all brain tumor types. Most therapies rarely provide clinically meaningful outcomes in the treatment of GBM. Although antibody-drug conjugates (ADCs) are promising anticancer drugs, no ADCs have been clinically successful for GBM, primarily because of poor blood-brain barrier (BBB) penetration. Here, we report that ADC homogeneity and payload loading rate are critical parameters contributing to this discrepancy. Although both homogeneous and heterogeneous conjugates exhibit comparable *in vitro* potency and pharmacokinetic profiles, the former shows enhanced payload delivery to brain tumors. Our homogeneous ADCs provide improved antitumor effects and survival benefits in orthotopic brain tumor models. We also demonstrate that overly drug-loaded species in heterogeneous conjugates are particularly poor at crossing the BBB, leading to deteriorated overall brain tumor targeting. Our findings indicate the importance of homogeneous conjugation with optimal payload loading in generating effective ADCs for intractable brain tumors.

## In brief

Most therapies rarely provide clinically meaningful improvements in glioblastoma multiforme (GBM) treatment. Anami et al. report that intravenous administration of homogeneous antibody-drug conjugates (ADCs) efficiently delivers payloads to brain tumors, leading to substantially improved tumor growth suppression. Their findings provide rational ADC design for effectively treating intractable brain tumors, including GBM.

## Graphical abstract

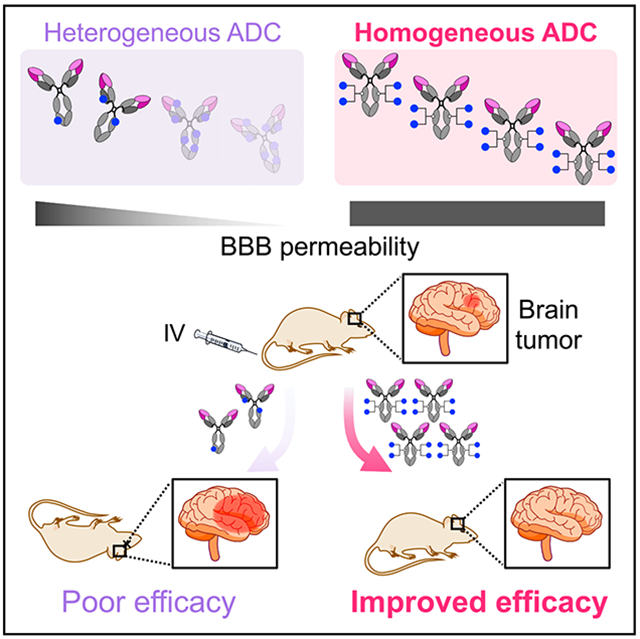

## INTRODUCTION

Glioblastoma multiforme (GBM) is the most aggressive brain tumor characterized by infiltrative growth to normal tissues, high proliferation rate, abundant angiogenesis, and intratumor and inter-patient heterogeneity (Inda et al., 2014; Parker et al., 2015; Shergalis et al., 2018). GBM has poorer survival rates than all other brain tumors (median survival time: 15–16 months) (Chinot et al., 2014; Stupp et al., 2005, 2009) because of quick relapse after standard therapy, namely, surgical removal in combination with radiation therapy, chemotherapy using temozolomide, and/or tumor-treating fields. Deep infiltration of GBM into normal brain tissues makes complete surgical resection of tumor lesions a challenging task. Although surgery is a proven option for primary GBM, its clinical benefit for patients with relapsed GBM remains unvalidated (Weller et al., 2014). To improve patients’ survival and quality of life, effective systemic therapies that can complement other treatment options are urgently needed.

Antibody-drug conjugates (ADCs) are an emerging class of chemotherapeutic agents consisting of tumor-targeting monoclonal antibodies (mAbs) with highly cytotoxic payloads attached through chemical linkers. Twelve ADCs have been approved by the US Food and Drug Administration (FDA) (Dhillon, 2018; Drago et al., 2021; Mullard, 2021), and more than 100 ADCs are currently in clinical trials (Chau et al., 2019). Despite the success in the management of other cancers, ADCs have not yet shown remarkable clinical outcomes in patients with GBM. Three ADCs have advanced to clinical trials for GBM therapy: depatuxizumab mafodotin (Depatux-M or ABT-414) (Phillips et al., 2016), ABBV-221 (Phillips et al., 2018), and AMG-595 (Hamblett et al., 2015). These ADCs target epidermal growth factor receptor (EGFR) and its active mutant EGFR variant III (EGFRvIII), which are signature receptors expressed in a subset of GBM tumors (Brennan et al., 2013). Unfortunately, these studies have been terminated or discontinued (Newman, 2019; Rosenthal et al., 2019; Van Den Bent et al., 2020). No survival benefit was confirmed in a phase 3 trial evaluating Depatux-M in patients with newly diagnosed GBM (Van Den Bent et al., 2020). In a preclinical study, ABBV-221 demonstrated greater treatment efficacy than could be achieved with Depatux-M; however, a phase 1 study has raised safety concerns (Newman, 2019). The development of AMG-595 was discontinued on completion of a phase 1 study because of limited efficacy. Unlike other solid tumors, efficient mAb delivery to the brain is particularly challenging because of the blood-brain barrier (BBB), a tightly constituted endothelial cell border restricting the influx of large molecules from the vasculature to the brain parenchyma (Abbott et al., 2010; Banks, 2016). Therefore, to establish ADC-based GBM therapy as a practical clinical option, identifying and optimizing molecular parameters that negatively influence BBB permeability, therapeutic efficacy, and safety profiles are critically important.

Herein, we report that ADC homogeneity plays a critical role in payload delivery to intracranial brain tumors. We demonstrate that homogeneous ADCs elicit improved antitumor activity in intracranial brain tumor-bearing mouse models compared with heterogeneous variants prepared by stochastic cysteine-maleimide or lysine-amide coupling. We also show using mouse models how homogeneous conjugation at an optimal drug-to-antibody ratio (DAR) improves efficiency in payload delivery to intracranial GBM tumors, leading to substantially extended survival. These findings suggest that ensuring ADC homogeneity is a crucial step to achieving clinically meaningful treatment outcomes in brain tumors, including GBM.

## RESULTS

### Construction of anti-EGFR ADCs with varied homogeneity

**Figure 1.**
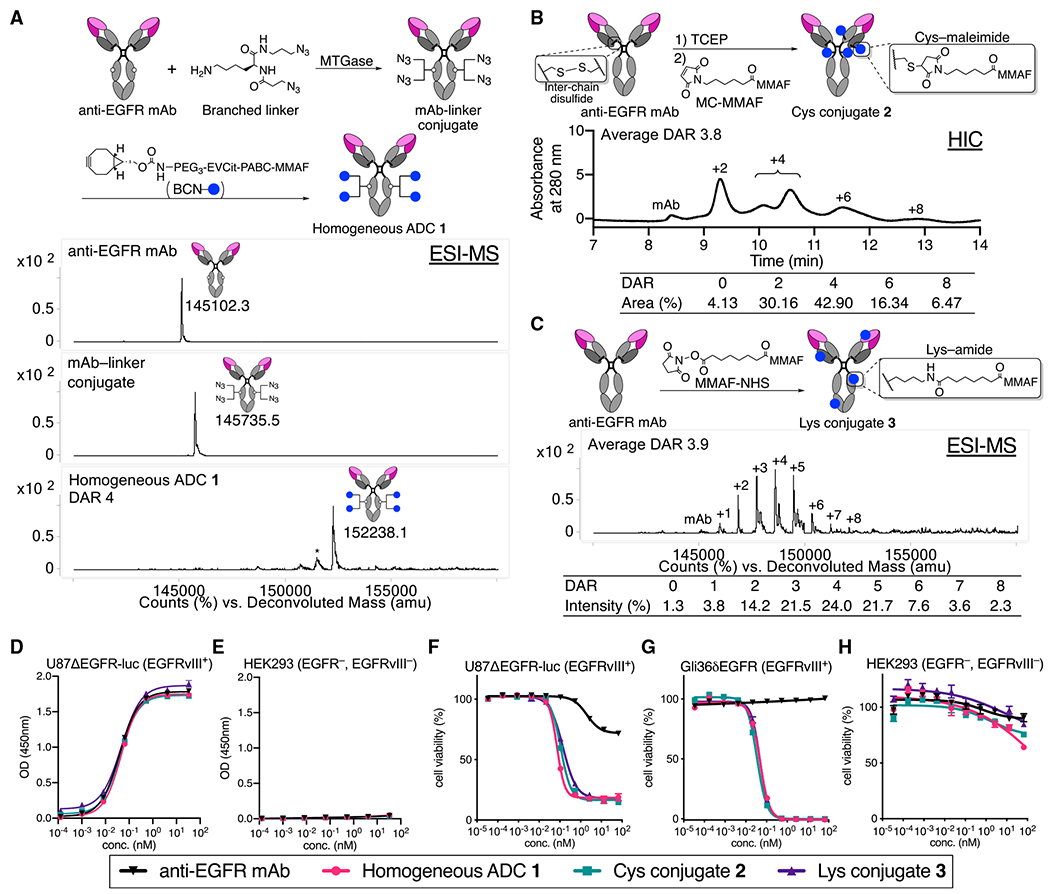
Construction, characterization, and *in vitro* evaluation of anti-EGFR ADCs (A) Preparation and electrospray ionization mass spectrometry (ESI-MS) analysis of homogeneous ADC **1**. Top panel: N88A/N297A anti-EGFR mAb (cetuximab mutant). Middle panel: mAb-linker conjugate. Bottom panel: homogeneous ADC **1**. Asterisk (*) indicates a fragment ion detected in ESI-MS analysis. (B) Preparation and HIC analysis of Cys conjugate **2**. (C) Preparation and ESI-MS analysis of Lys conjugate **3**. (D and E) Cell-based ELISA in U87ΔEGFR-luc (EGFRvIII^+^) and HEK293 (EGFR^−^, EGFRvIII^−^) cells. (F–H) Cell-killing potency in U87ΔEGFR-luc, Gli36δEGFR (EGFRvIII^+^), and HEK293 (mean values ± SEM, n = 3). Concentrations are based on the antibody dose without normalizing to each DAR.

We have previously established click chemistry-empowered branched linkers for installing two identical or different payloads onto a single aglycosylated antibody in a site-specific and quantitative manner (Anami and Tsuchikama, 2020; Anami et al., 2017; Yamazaki et al., 2021). We have also developed the glutamic acid-valine-citrulline (EVCit) cleavable linker enabling the intracellular release of payloads in a traceless fashion while minimizing premature linker degradation in human and mouse plasma (Anami et al., 2018). Using these technologies, we set out to construct a homogeneous ADC targeting both EGFR and EGFRvIII (Figure 1A). We used cetuximab (EliLilly, 2004) with N88A and N297A double mutations as a parent mAb. The N88A/N297A double mutations remove two N-glycans on the side chains of asparagine 88 within the Fab moiety and asparagine 297 within the Fc moiety (Giddens et al., 2018). N-glycan removal abrogates immune responses derived from interactions with Fcγ receptors expressed in immune cells, which can minimize undesired systemic toxicity or inflammatory response (Herbst et al., 2020; White et al., 2020). Atezolizumab (TECENTRIQ, anti-programmed death-ligand 1 [PD-L1] mAb) is a recent example with an N297A mutation approved by the FDA.

We began the ADC construction by installing branched diazide linkers site specifically onto glutamine 295 (Q295) within the parent N88A/N297A anti-EGFR mAb using microbial transglutaminase (MTGase) (Anami and Tsuchikama, 2020) (Figure 1A). In parallel, we synthesized a payload module consisting of bicyclo [6.1.0]nonyne (BCN; reaction handle for following strain-promoted azide-alkyne click reaction), EVCit (cathepsin-responsive cleavable sequence), *p*-aminobenzyloxycarbonyl (PABC) spacer, and monomethyl auristatin F (MMAF). Finally, the click reaction between the payload module BCN-EVCit-PABC-MMAF and the azide groups within the mAb-branched linker quantitatively afforded homogeneous anti-EGFR ADC **1** with a DAR of 4 (Figures 1A and S1A). Using the same parent anti-EGFR mAb, we also prepared two heterogeneous variants that resemble the structure of Depatux-M (Cys conjugate **2**) (Phillips et al., 2016) or the conjugation modality of AMG-595 (Lys conjugate **3**) (Hamblett et al., 2015). For the preparation of Cys conjugate **2**, non-cleavable maleimidocaproyl MMAF (MC-MMAF) was installed by partial disulfide bond reduction and following cysteine-maleimide alkylation. We confirmed by hydrophobic interaction chromatography (HIC) analysis that Cys conjugate **2** consisted of DAR 0, 2, 4, 6, and 8 species (average DAR: 3.8; Figure 1B). To prepare Lys conjugate **3**, we synthesized and used non-cleavable MMAF-*N*-hydroxysuccinimide (NHS) ester for lysine coupling. Mass spectrometry analysis revealed that this heterogeneous conjugate consisted of multiple products with DARs ranging from 0 to 8 (average DAR: 3.9; Figure 1C).

### Cysteine-maleimide conjugation does not impair EGFR-specific potency *in vitro* but reduces long-term stability

Size-exclusion chromatography (SEC) analysis revealed that all ADCs generated predominantly existed in the monomer form (Figure S1B). These ADCs were also tested for long-term stability under physiological conditions (pH 7.4, 37°C, 28 days). We observed no significant degradation or aggregation for homogeneous ADC **1** and Lys conjugate **3** (Figures S1C–S1F). In contrast, Cys conjugate **2** underwent fragmentation or partial dissociation of the heavy and light chains. These results suggest that both MTGase-mediated homogeneous conjugation and lysine coupling offer higher thermal stability than can be achieved by cysteine-maleimide conjugation.

Next, we assessed antigen-specific binding of the ADCs by cell-based ELISA (Figures 1D and 1E; Table S1). All ADCs showed binding affinities for EGFRvIII-positive U87ΔEGFR-luc cells (K_D_: 0.044–0.047 nM) comparable with that of the unmodified N88A/N297A cetuximab (K_D_: 0.039 nM). In addition, none of the ADCs bound to EGFR-negative HEK293 cells. These results demonstrate that the ADCs retained their binding affinity and specificity regardless of conjugation methods. We also tested these conjugates for cell-killing potency in U87ΔEGFR-luc, Gli36δEGFR, and HEK293 cells (Figures 1F–1H; Table S2). All DAR 4 ADCs showed comparable potency in the EGFRvIII-positive GBM cells, but not in HEK293 cells. This result is in line with previous reports demonstrating that MMAF ADCs can exert pM-level potency with or without a cleavable linker (Deonarain et al., 2014; Doronina et al., 2006).

### The homogeneous anti-EGFR ADC exerts significantly improved therapeutic efficacy in orthotopic mouse models of GBM

**Figure 2.**
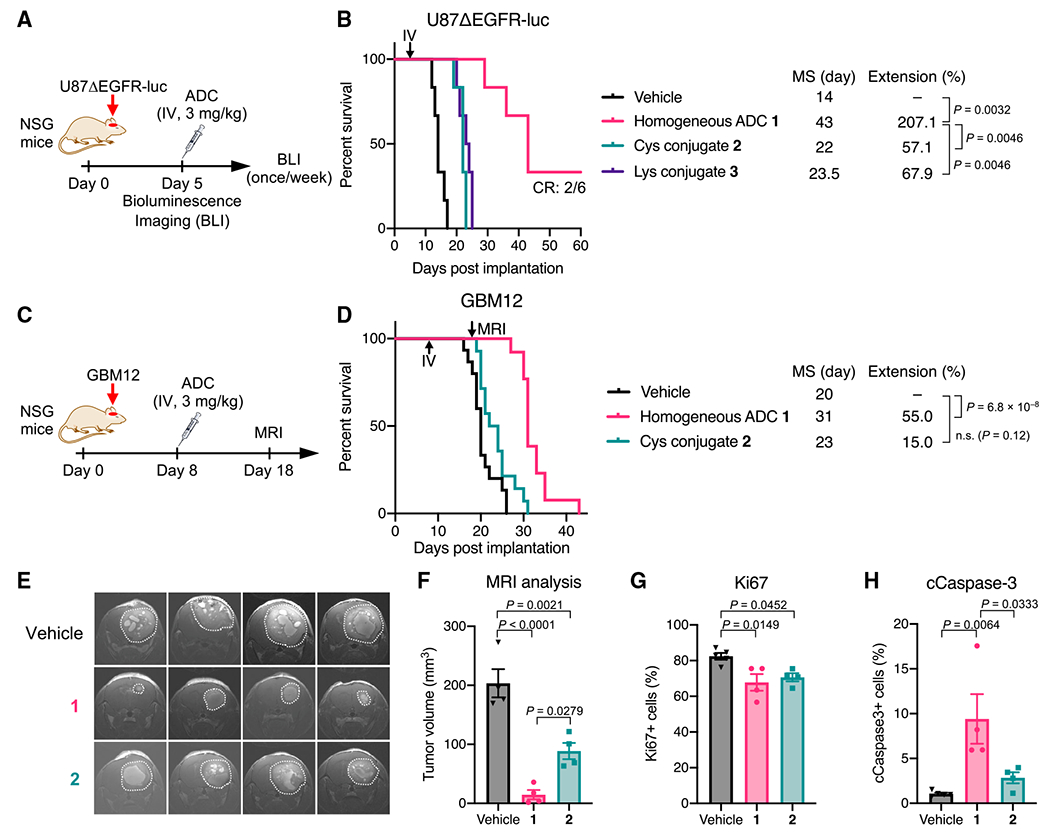
ADC homogeneity enhances therapeutic efficacy in orthotopic GBM mouse models (A) Study schedule in the U87ΔEGFR-luc xenograft model (male and female NSG mice). (B) Survival curves in the U87ΔEGFR-luc model (n = 6/group). p values were calculated using a log rank test with a Bonferroni correction. (C) Study schedule in the GBM12 PDX model (male and female NSG mice). (D) Survival curves in the GBM12 model (n = 15 for vehicle; n = 14 for ADCs). p values were calculated using a log rank test with a Bonferroni correction. (E) Coronal magnetic resonance (MR) images on day 18 (n = 4). Tumor lesions are indicated with white dots. (F) Estimated tumor volume by MR image-based quantification (mean values ±SEM; n = 4). One-way ANOVA with a Tukey-Kramer post hoc test was used for statistical analysis. (G and H) Populations of Ki67-positive cells (G) and cCaspase-3-positive cells (H) in the GBM12 tumors harvested at the terminal stage (mean values ±SEM; n = 5 for vehicle, n = 4 for ADCs). One-way ANOVA with a Tukey-Kramer post hoc test was used for statistical analysis.

To evaluate the *in vivo* antitumor activity of the three anti-EGFR ADCs, we first performed a treatment study using a cell line-derived xenograft model of human GBM. To gain clinically translatable insights into the influence of conjugation modality on brain tumor targeting, we used intracranially implanted models instead of subcutaneous models. NOD *scid* gamma (NSG) mice bearing orthotopic U87ΔEGFR-luc tumors (5 days post-implantation, tumor volume: 8.69 ± 1.80 mm^3^; Figure S2A) were injected intravenously with a single dose of each ADC at 3 mg/kg (Figure 2A). No acute toxicity associated with ADC administration was observed in either group over the course of the study (Figure S2B). The short survival time observed for the untreated group (median survival: 14 days; Figure 2B) demonstrates the extremely aggressive growth of this GBM model. Homogeneous ADC **1** exerted remarkable antitumor activity with statistically significant survival benefits (median survival: 43 days; p = 0.0032). In addition, two of six mice survived at the end of the study (day 60) with no detectable bioluminescence signal from implanted tumors (Figure S2C), indicating that these two mice achieved complete remission (CR). In contrast, the heterogeneous conjugates exhibited limited therapeutic effects with marginally increased median survival times (22 days for Cys conjugate **2** and 23.5 days for Lys conjugate **3**), which were inferior to that provided by homogeneous ADC **1** (p = 0.0046). Indeed, all mice in these two groups died or reached the pre-defined humane endpoint by the end of the study (Figure 2B). This result is in contrast with our observation that ADCs **1–3** showed comparable *in vitro* cell-killing potency in U87ΔEGFR-luc cells (Figure 1F).

Next, we sought to use a patient-derived xenograft (PDX) tumor model of GBM. PDX models maintain pathohistological and genetic properties of original tumors, as well as therapeutic responses to anti-cancer treatments, allowing to obtain clinically relevant and translatable data (Hidalgo et al., 2014). We used GBM12, a PDX model overexpressing wild-type EGFR (Sarkaria et al., 2006). A study has shown that GBM12 tumors show heterogeneous BBB disruption, meaning that some GBM12 tumor cells are likely protected by an intact BBB (Parrish et al., 2015). Before initiating an *in vivo* assessment, homogeneous ADC **1** and heterogeneous Cys conjugate **2** were evaluated for *in vitro* cell-killing potency. Both ADCs efficiently killed GBM12 cells with comparable pM-level EC_50_ values (Figure S3A). Next, we investigated whether homogeneous conjugate **1** also showed a greater treatment effect *in vivo* than could be achieved by Cys conjugate **2**. NSG mice bearing intracranial GBM12 tumors (8 days post-implantation, tumor volume: 2.52 ± 0.68 mm^3^; Figure S3B) were injected intravenously with a single dose of either conjugate at 3mg/kg (Figure 2C). No acute toxicity was observed in either group over the course of the study (Figure S3C). Homogeneous ADC **1** effectively suppressed tumor growth with a statistically significant survival benefit (median survival: 31 days, p = 6.8 × 10^−8^), whereas Cys conjugate **2** showed a marginal therapeutic effect (median survival: 23 days, p = 0.12; Figure 2D). Magnetic resonance imaging (MRI) on day 18 revealed that the tumors treated with homogeneous ADC **1** (14.71 ± 7.90 mm^3^) were markedly smaller than the untreated ones (203.46 ± 23.81 mm^3^, p < 0.0001; Figures 2E and 2F). Cys conjugate **2** also inhibited tumor growth (88.63 ± 13.71 mm^3^) but less effectively than homogeneous ADC **1** (p = 0.0279). To investigate how each ADC influenced cell proliferation and apoptosis, we performed immunohistochemistry analysis of brain tissues harvested from each group at the terminal stage (vehicle: 20–26 days, ADC **1**: 30–35 days, Cys conjugate **2**: 24–31 days; Figures S3D–S3G). About 80% of cells were Ki67 positive in the vehicle-treated group, while about 70% of cells were Ki67 positive in both ADC-treated groups (Figure 2G). This result indicates that antiproliferative effects by both ADCs declined to similar levels at the terminal stage. In contrast, the population of cleaved caspase-3 (cCaspase-3)-positive cells in the tumors treated with ADC **1** (9.4% ± 2.8%) was significantly higher than that in the tumors treated with vehicle (1.1% ± 0.1%, p = 0.0064) or Cys conjugate **2** (2.8% ± 0.6%, p = 0.0333; Figure 2H), suggesting that homogeneous ADC **1** induced apoptosis more effectively than heterogeneous ADC **2** over the course of the study. Given that the histopathology analysis was performed at the terminal stage of each group, more significant differences in Ki67 and cCaspase-3 levels could have been observed at the same time point in the early stage. Collectively, these results demonstrate that homogeneous ADC **1** can eradicate intracranial GBM tumors more efficiently than its heterogeneous variants.

### Homogeneity also improves *in vivo* therapeutic efficacy of other ADCs for EGFRvIII- and HER2-positive brain tumors

**Figure 3.**
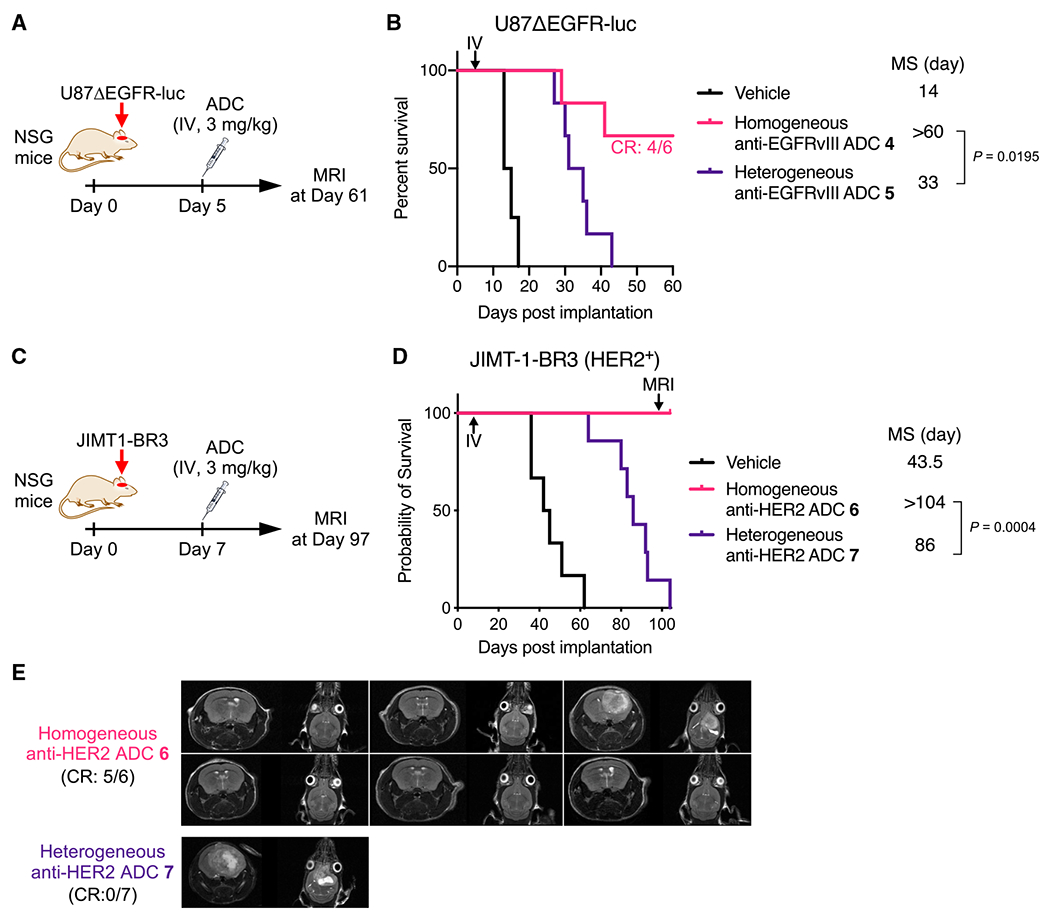
Homogeneous depatuxizumab- and trastuzumab-based ADCs show enhanced therapeutic efficacy in orthotopic brain tumor mouse models (A) Study schedule in the orthotopic U87ΔEGFR-luc xenograft mouse model (male and female NSG mice). (B) Survival curves in the U87ΔEGFR-luc model (n = 4 for vehicle; n = 6 for homogeneous ADC **4** and heterogeneous conjugate **5**). A log rank test was used for statistical analysis. CR in the homogeneous ADC **4** group: 4/6. (C) Study schedule for the intracranially implanted JIMT-1-BR3 tumor mouse model (female NSG mice). (D) Survival curves in the JIMT-1-BR3 model (n = 6 for vehicle and homogeneous ADC **6**; n = 7 for heterogeneous ADC **7**). A log rank test was used for statistical analysis. (E) Coronal and sagittal MR images of the intracranial JIMT-1-BR3 tumor-bearing mice on day 97. CR: 5/6 (homogeneous ADC **6**) and 0/7 (ADC **7**).

To generalize our findings, we tested other homogeneous ADCs for treatment efficacy in orthotopic brain tumor models. Homogeneous anti-EGFRvIII ADC **4** (DAR 4) and heterogeneous variant **5** (Lys conjugate, average DAR: 4.7) were constructed from N297A depatuxizumab, the parent mAb of Depatux-M (Phillips et al., 2016) (Figure S4A). Both ADCs showed comparable cell-killing potency in U87ΔEGFR-luc cells (Figure S4B). Subsequently, NSG mice bearing intracranial U87ΔEGFR-luc tumors were treated with a single dose of each ADC at 3 mg/kg at 5 days post-implantation (Figure 3A). Homogeneous ADC **4** showed a remarkable survival benefit (median survival: >60 days), and four of six mice treated survived over the course of the study. In addition, MRI on day 61 showed no detectable brain tumor lesion in these survivors, indicating complete remission (Figure S4C). In contrast, heterogeneous variant **5** extended median survival time (33 days) less significantly than homogeneous ADC **4** (p = 0.0195; Figure 3B).

Next, we performed similar *in vitro* and *in vivo* studies using a HER2-positive brain tumor model. Brain metastasis is observed in 25%–50% of patients with advanced HER2-positive breast tumors (Zimmer et al., 2020), representing a difficult-to-treat population. We prepared anti-HER2 homogeneous ADC **6** (DAR 4) and a heterogeneous variant (Lys conjugate **7**, average DAR: 4.2) from N297A trastuzumab and evaluated their cell-killing potency in HER2-positive brain-tropic JIMT-1-BR3 cells (Palmieri et al., 2014) (Figures S5A and S5B). Both ADCs efficiently killed JIMT-1-BR3 cells with comparable half maximal effective concentration (EC_50_) values (Figure S5B). Subsequently, NSG mice bearing intracranial JIMT-1-BR3 tumors (7 days post-implantation, tumor volume: 2.33 ± 0.29 mm^3^; Figure S5C) were injected intravenously with each ADC at 3 mg/kg (Figure 3C). All mice treated with homogeneous ADC **6** survived over the course of the study, while the median survival times without treatment and with treatment by heterogeneous ADC **7** were 43.5 and 86 days, respectively (Figure 3D). MRI analysis on day 97 revealed that brain tumor lesions were detected in one of six mice treated with homogeneous ADC **6** (CR: 5/6) and in the survivor mouse treated with heterogeneous ADC **7** (CR: 0/7) (Figure 3E). Collectively, these findings strongly support our hypothesis that the use of homogeneous ADCs can lead to significantly improved treatment outcomes in a broad range of brain tumors.

### Clearance and linker stability in circulation are not the primary factors reducing the efficiency in payload delivery to brain tumors

**Figure 4.**
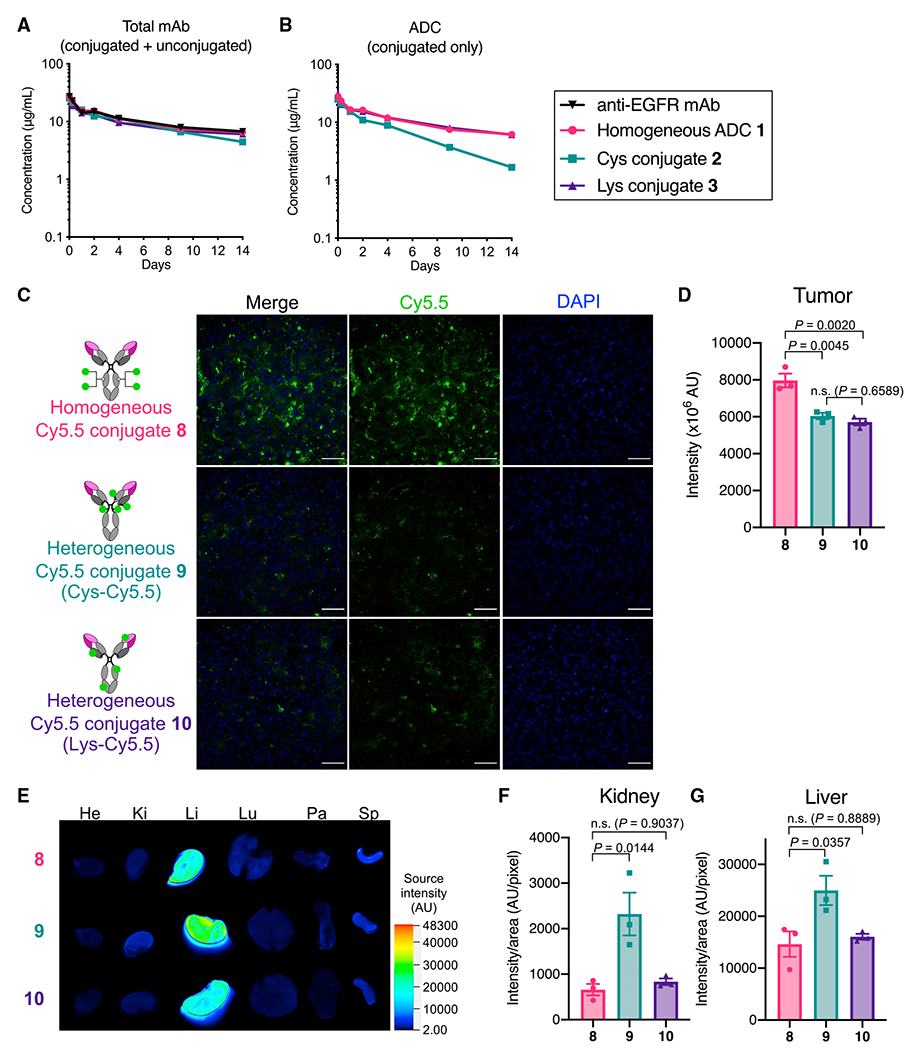
Promoted clearance and premature payload loss in circulation are not the primary factors attenuating the brain-tumor-targeting efficiency of ADCs (A and B) PK of unmodified N88A/N297A anti-EGFR mAb and ADCs **1–3** in female CD-1 mice (n = 3). Concentrations of total antibody (both conjugated and unconjugated, A) and ADC (intact ADC-equivalent dose, B) were determined by sandwich ELISA (mean values ± SEM). (C) Fluorescence images of brain tumor tissues harvested 48 h after injecting each Cy5.5 conjugate to male and female NSG mice bearing orthotopic U87ΔEGFR tumors (representative of three independent experiments, scale bar: 50 μm). (D) Semi-quantification of the Cy5.5 signal detected in the brain tumor tissues. Three regions of interest (ROIs) were randomly selected in each tissue sample to calculate signal intensity (mean values ± SEM). One-way ANOVA with a Tukey-Kramer post hoc test was used for statistical analysis. (E) *Ex vivo* fluorescence images of the other organs (He, heart; Ki, kidney; Li, liver; Lu, lung; Pa, pancreas; Sp, spleen). A representative result of three independent experiments is shown. (F and G) Semi-quantification of the Cy5.5 signal detected in the kidneys and liver (mean values ± SEM). One-way ANOVA with a Tukey-Kramer post hoc test was used for statistical analysis.

To understand how the antibody-drug conjugation modality impacts overall therapeutic efficacy in brain tumors, we set out to assess *in vivo* pharmacokinetic (PK) profiles of selected ADCs. Anti-EGFR ADCs **1–3** and the parental anti-EGFR mAb were intravenously administered into CD-1 mice at 3 mg/kg. The concentrations of total mAb and ADC in plasma were then determined by sandwich ELISA. In total mAb analysis, homogeneous ADC **1** and Lys conjugate **3** showed half-lives at the elimination phase (t_1/2β_ = 9.8 days, ADC **1**; t_1/2β_ = 10.4 days, Lys conjugate **3**) comparable with that of the unmodified mAb (10.9 days), whereas Cys conjugate **2** showed a slightly decreased half-life (t_1/2β_ = 7.8 days; Figure 4A; Table S3). We found that Cys conjugate **2** showed thermal instability after a 28-day incubation under physiological conditions probably because of partly cleaved interchain disulfide bonds (Figure S1E). This instability may account for the increased clearance rate. In payload-based ELISA, no significant reduction in half-lives was observed for homogeneous ADC **1** (t_1/2β_ = 8.6 days) or Lys conjugate **3** (t_1/2β_ = 8.9 days), indicating that there was almost no premature release of MMAF during circulation (Figures 4B and S6; Table S3). In contrast, the intact ADC-equivalent concentration of Cys conjugate **2** declined at a faster rate (t_1/2β_ = 4.2 days), indicating that the conjugated MMAF was partly lost. Previous reports have shown that cysteine-containing serum proteins such as albumin promote dissociation of cysteine-maleimide linkage within ADCs through a thiol exchange reaction, leading to partial deconjugation of payloads in circulation (Lyon et al., 2014; Tumey et al., 2014).

As demonstrated above, promoted clearance and payload deconjugation may partly account for the poor treatment efficacy observed for Cys conjugate **2** in the orthotopic GBM models. However, these factors are likely irrelevant to the inferior efficacy observed for the lysine conjugates, which were designed not to show thermal instability or undergo deconjugation in circulation. To uncover other contributing factors, we performed a biodistribution study using the orthotopic U87-ΔEGFR-luc xenograft mouse model. We chose a sulfo-Cy5.5 dye (termed Cy5.5 hereafter) as a surrogate of MMAF for the following reason: both Cy5.5 and MMAF are hydrophilic molecules and can stay inside the cell because of a lack of cell permeability. The following fluorescent dye conjugates were prepared from the N88A/N297A cetuximab: homogeneous Cy5.5 conjugate **8** (degree of labeling [DOL]: 4) and two heterogeneous Cy5.5 conjugates by cysteine-maleimide coupling (Cys-Cy5.5 conjugate **9**, average DOL: 4.1) and lysine coupling (Lys-Cy5.5 conjugate **10**, average DOL: 4.1; see supplemental information for details). In all cases, Cy5.5 was incorporated as a payload surrogate into the parent mAb with the same linkers and conjugation chemistries that were used to prepare corresponding ADCs. Orthotopic U87ΔEGFR-luc tumor-bearing NSG mice (5 days post-implantation) were administered intravenously with each dye conjugate at 3 mg/kg. Fluorescence imaging of the harvested brains revealed that homogeneous Cy5.5 conjugate **8** accumulated in the brain tumors more effectively than heterogeneous conjugates **9** (p = 0.0045) and **10** (p = 0.0020; Figures 4C and 4D). We also confirmed that all Cy5.5 conjugates were specifically accumulated in the brain tumor region (Figures S6D–S6F). We did not see a significant difference in intracranial U87ΔEGFR-luc tumor-targeting ability between Cys conjugate **9** and Lys conjugate **10**. We also confirmed in a separate biodistribution study that the cathepsin-responsive cleavage EVCit linker did not significantly contribute to the enhanced accumulation in U87ΔEGFR-luc tumors (Figures S6G). Cys-Cy5.5 conjugate **9** showed an increased fluorescent signal in the kidneys and liver compared with homogeneous Cy5.5 conjugate **8** (kidneys: p = 0.0144, liver: p = 0.0357), probably because of partial deconjugation of the maleimide-Cy5.5 modules in circulation and following hepatic and renal clearance (Figures 4E–4G). However, we did not observe such increased liver and kidney accumulation for Lys-Cy5.5 conjugate **10**. Taken together, these findings suggest that promoted clearance of conjugated payloads and linker instability are not the primary factors attenuating the brain-tumor-targeting efficiency.

### Homogeneous conjugation enables efficient payload delivery to intracranial tumors for days

**Figure 5.**
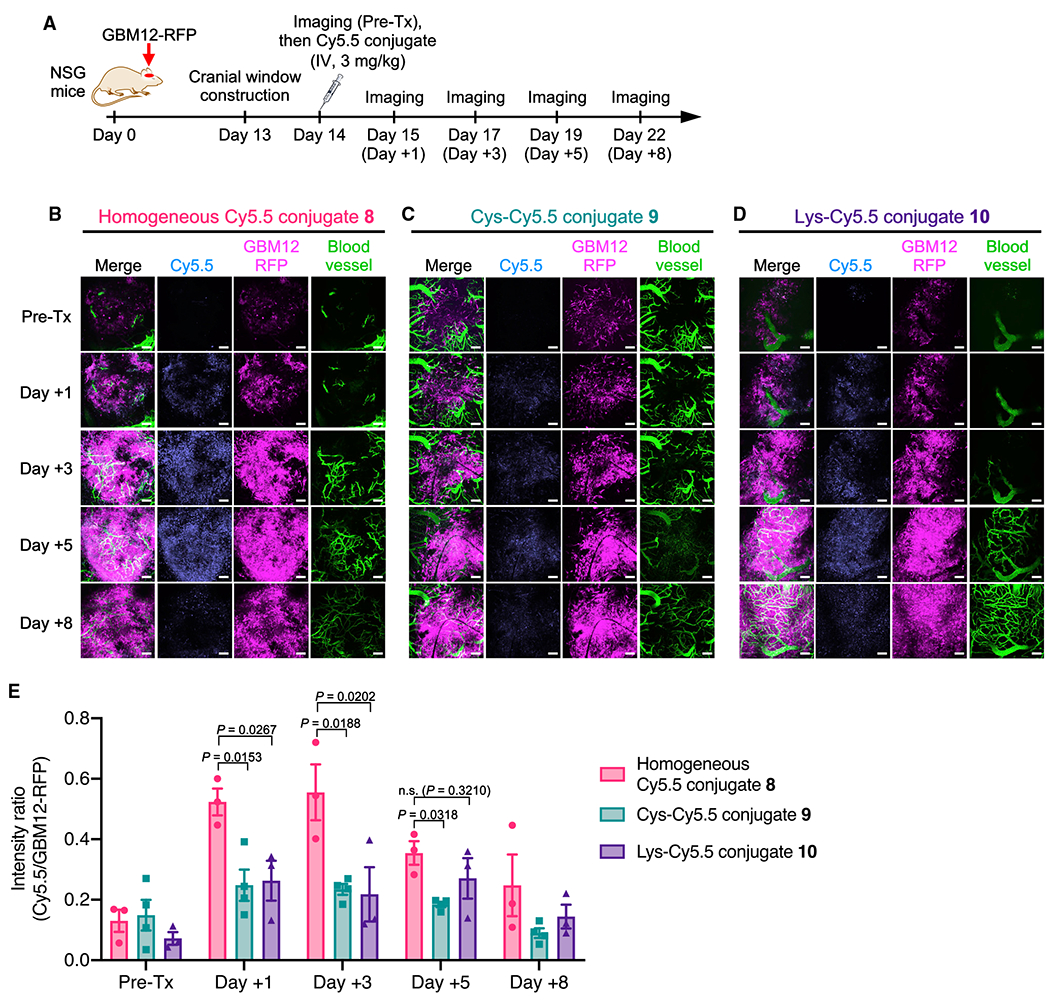
Homogeneous conjugation allows for enhanced payload delivery to orthotopically xenografted GBM tumors for several days (A) Study schedule for intravital imaging (male NSG mice). FITC-conjugated dextran was injected right before each imaging session to visualize the brain microvasculature. (B–D) Intravital images of GBM12-RFP tumors treated with homogeneous Cy5.5 conjugate **8** (n = 3, B), Cys-Cy5.5 conjugate **9** (n = 4, C), and Lys-Cy5.5 conjugate **10** (n = 3, D). Representative images at each time point are shown. Scale bar: 100 μm. (E) Normalized Cy5.5 intensity (Cy5.5 signal/GBM12-RFP signal, mean values ± SEM, n = 3 for conjugates **8** and **10**; n = 4 for conjugate **9**). One-way ANOVA with a Dunnett’s post hoc test (control: homogeneous conjugate **8**) was used for statistical analysis. Pre-Tx, pre-treatment.

We performed longitudinal intravital imaging to clarify spatiotemporal changes in the accumulation of payloads in brain tumors. GBM12 cells that stably express Red Fluorescent Protein (GBM12-RFP) were implanted into NSG mice intracranially, and either Cy5.5 conjugate **8**, **9**, or **10** was administered intravenously at 3 mg/kg at 14 days post-implantation (Figure 5A). As demonstrated by the increasing RFP signals, the implanted tumors continued to grow throughout the study (Figures 5B–5D). To offset the intragroup and intergroup variances derived from tumor growth, the Cy5.5 signal intensity was normalized to the RFP signal intensity at each time point. The intratumor concentrations of the three conjugates peaked around day 3 post-administration and then declined over time (Figure 5E). Notably, homogeneous conjugate **8** accumulated in the orthotopic GBM12 tumors more significantly and persistently than heterogeneous variants **9** and **10**; the statistically significant enhancement was observed for up to 5 days (Figure 5E). Overall, these results suggest that homogeneous conjugation allows intravenously administered antibody conjugates to target brain tumors with enhanced payload delivery efficiency and durability.

### High-DAR components in heterogeneous MMAF ADCs target brain tumors less efficiently than low-DAR components

**Figure 6.**
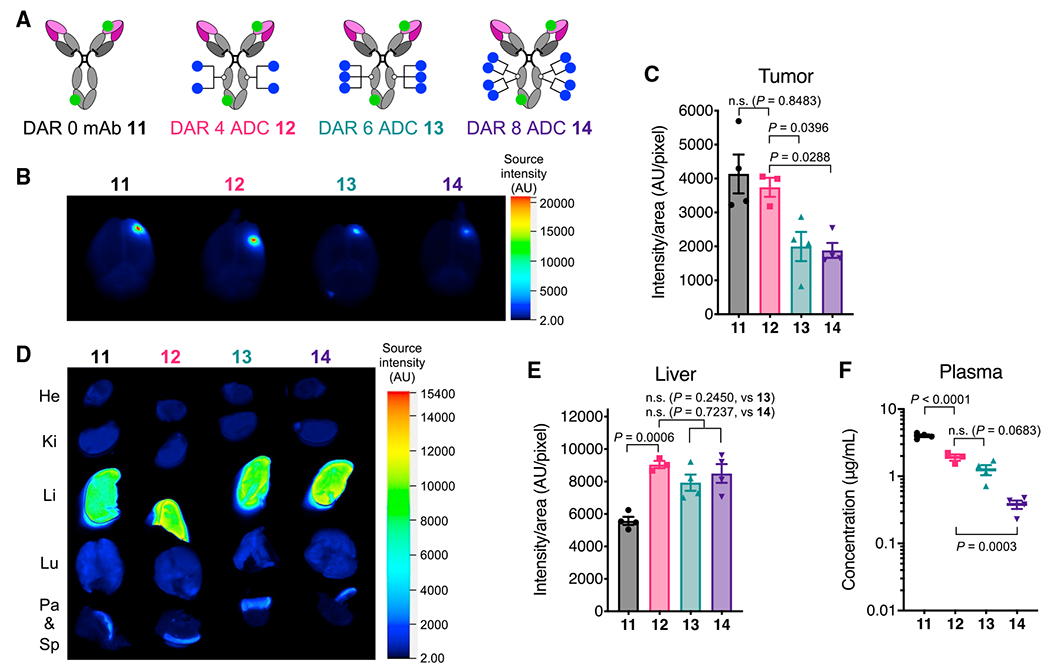
High-DAR components in heterogeneous ADCs target brain tumors less efficiently than components with optimal or low DAR (A) The structures of fluorescently labeled anti-EGFRvIII ADCs equipped with MMAF (blue circle) at DARs of 0, 4, 6, and 8. Cy5.5 (green circle) was conjugated by lysine coupling at DOL of 2.3–2.5. (B) Representative *ex vivo* fluorescence images of whole brains harvested from male NSG mice bearing orthotopic U87ΔEGFR-luc tumors 48 h after intravenous injection of each fluorescent ADC (n = 3 for DAR 4 ADC **12**; n = 4 for all other groups). (C) Semi-quantification of the Cy5.5 signal derived from the tumor lesions in the whole brains (mean values ± SEM, n = 3 for DAR 4 ADC **12**; n = 4 for all other groups). One-way ANOVA with a Dunnett’s post hoc test (control: DAR 4 ADC **12**) was used for statistical analysis. (D) Representative *ex vivo* fluorescence images of other major organs (n = 3 for DAR 4 ADC **12**; n = 4 for all other groups). (E) Semi-quantification of the Cy5.5 signal detected in the liver (mean values ± SEM; n = 3 for DAR 4 ADC **12**, n = 4 for all other groups). One-way ANOVA with a Dunnett’s post hoc test (control: DAR 4 ADC **12**) was used for statistical analysis. (F) Concentrations of total antibody in plasma collected right before cardiac perfusion (mean values ± SEM; n = 3 for DAR 4 ADC **12**, n = 4 for all other groups). One-way ANOVA with a Dunnett’s post hoc test (control: DAR 4 ADC **12**) was used for statistical analysis.

Finally, we set out to clarify underlying mechanisms attenuating the brain-tumor-targeting efficiency of heterogeneous ADCs. To investigate how each DAR component could affect biodistribution profiles, we prepared depatuxizumab-based MMAF ADCs with DARs of 4, 6, and 8 using our branched linkers and non-cleavable BCN-MMAF. The parent N297A depatuxizumab was used as a DAR 0 control. These anti-EGFRvIII mAbs and conjugates were then labeled with Cy5.5 NHS ester at DOL of 2.3–2.5 to afford mAb **11** and ADCs **12–14** (Figure 6A; see supplemental information). Cy5.5 was installed directly onto the mAb scaffold so that the fluorescent signal would represent the localization of the entire ADC structure. The fluorescent conjugates (3 mg/kg) were injected intravenously into NSG mice bearing orthotopic U87ΔEGFR-luc tumors on day 5 after tumor implantation. After blood collection and cardiac perfusion at 48 h, major organs were harvested for fluorescence imaging. DAR 0 mAb **11** and DAR 4 ADC **12** showed similar levels of brain tumor accumulation (Figures 6B and 6C). In contrast, compared with DAR 4 ADC **12**, markedly attenuated brain tumor accumulation was observed for DAR 6 ADC **13** (p = 0.0396) and DAR 8 ADC **14** (p = 0.0288). Although ADCs **12–14** accumulated in the liver more significantly than DAR 0 mAb **11**, the degrees of liver accumulation and biodistribution patterns of these ADCs were similar and irrespective of DAR (Figures 6D and 6E). In addition, the concentrations of DAR 4 and 6 ADCs **12** and **13** in blood were in a similar range and slightly below that of DAR 0 mAb **11** (Figure 6F). DAR 8 ADC **14** underwent accelerated clearance from the circulation probably because of greatly increased hydrophobicity. Collectively, these results demonstrate that high-DAR components comprising a given heterogeneous ADC can show limited brain tumor targeting compared with components with optimal or low DARs, leading to reduced overall payload delivery efficiency.

## DISCUSSION

We have investigated how ADC homogeneity impacts therapeutic efficacy and survival extension in orthotopic brain tumor models. We tested our homogeneous ADCs and heterogeneous variants prepared by conventional lysine or cysteine coupling for antiproliferative effect against several brain tumor cells. *In vitro*, all DAR-matched ADCs showed comparable antigen-specific binding and cell-killing potency irrespective of ADC homogeneity or conjugation method. In addition, we did not observe a significant difference in cell-killing potency between our cleavable ADCs and non-cleavable variants. This observation is reasonable because MMAF is a payload that can exert a cell-killing effect regardless of linker cleavability (Doronina et al., 2006). However, we obtained contrasting results *in vivo*; all homogeneous ADCs exerted far better survival benefits in both cell line-derived xenograft and PDX orthotopic brain tumor models than could be achieved by corresponding heterogeneous variants, including a Depatux-M surrogate. Notably, a single dose of our homogeneous ADCs provided complete remission in the orthotopic U87ΔEGFR-luc (two of six mice by anti-EGFR ADC **1**; four of six mice by anti-EGFRvIII ADC **4**) and JIMT-1-BR3 models (five of six mice by anti-HER2 ADC **6**), whereas DAR-matched heterogeneous ADCs did not in either case. To delve into this discrepancy, we performed biodistribution studies using intracranially xenografted GBM models. Our data from these studies indicate that homogeneous conjugation at optimal DARs likely allows for enhanced and persistent payload accumulation into intracranial tumors over several days, leading to improved *in vivo* efficacy. We also confirmed that both cleavable and non-cleavable linkers allowed homogeneous anti-EGFR conjugates to deliver payloads to intracranial GBM tumors at similar levels. Collectively, these results demonstrate that ADC homogeneity is a critical factor determining therapeutic efficacy in brain tumors.

The question we asked next is how ADC homogeneity critically influences systemic payload delivery to brain tumors. Many studies have shown that homogeneous ADCs provide more favorable therapeutic effects in the treatment of other solid tumors than can be achieved by heterogeneous variants (Bryant et al., 2015; Junutula et al., 2008, 2010; Lhospice et al., 2015; Pillow et al., 2014). Nevertheless, the improvement in therapeutic efficacy observed in our study appears to be much more prominent compared with those cases. We think that blockage of drug influx by an intact BBB in and around brain tumors likely answers this question. The BBB was believed to be uniformly and significantly disrupted in GBM tumors. Contrary to this previous belief, recent preclinical and clinical studies have demonstrated that a measurable number of GBM cells, in particular ones near the growing edge of the infiltrative tumor area, exist behind an intact BBB or partially functional blood-tumor barrier (BTB) (Arvanitis et al., 2020; Kim et al., 2018; Marin et al., 2021; Sarkaria et al., 2018; van Tellingen et al., 2015). As such, GBM cells protected by an intact BBB are inaccessible to systemically administered ADCs. Recently, Marin et al. (2021) exhaustively validated heterogeneous BBB disruption in multiple PDX models of GBM, including GBM12. They also demonstrated that the intact BBB likely caused an uneven intracranial distribution of systemically administered Depatux-M, resulting in insignificant treatment outcomes in five of seven orthotopic PDX models. In contrast, they found that Depatux-M exerted remarkable therapeutic effects when tested in subcutaneous models of the same PDX tumors, in which the BBB did not constitute the tumor microenvironment. This report highlights the importance of testing ADCs for brain tumor treatment in clinically relevant orthotopic models rather than subcutaneous models.

**Figure 7.**
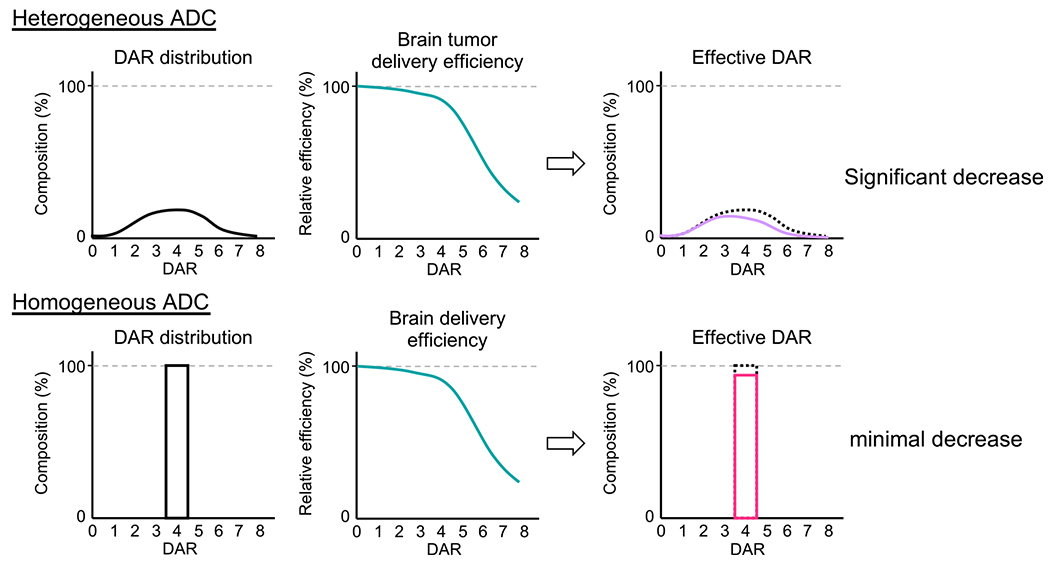
Reduction in effective DAR and payload dose is more prominent in heterogeneous ADCs than in homogeneous ADCs All values used in this figure are estimated values based on the data shown in Figures 1 and 6. Theoretical payload doses of heterogeneous and homogeneous ADCs with the same (average) DAR are equivalent if administered at the same mAb dose. However, high-DAR components in heterogeneous ADCs show poor brain tumor targeting, decreasing the effective DAR and payload dose. Such deterioration is marginal in the case of homogeneous ADCs, leading to improved payload delivery and overall efficacy.

Our findings and the report from Marin et al. (2021) led to a hypothesis that high-DAR species in heterogeneous ADCs cannot be efficiently delivered to intracranial tumors across the BBB compared with low-DAR species. Consequently, the effective DAR (i.e., DARs adjusted based on the brain-tumor-targeting efficiency of each DAR component relative to that of the unmodified mAb) and payload dose are considerably reduced (Figure 7). In contrast, homogeneous ADCs constructed at optimal DARs likely undergo only marginal impairment in brain tumor targeting, leading to a minimal reduction in effective payload dose. Indeed, our intravital imaging study showed that the difference in payload dose between heterogeneous and homogeneous ADCs could reach up to 2.5-fold. In general, ADC hydrophobicity increases in proportion to the degree of payload conjugation. As such, high-DAR ADCs have greater aggregation tendency compared with low-DAR ADCs (Beckley et al., 2013; Buecheler et al., 2018). In circulation, such multimolecular complexation may also occur with abundant proteins such as albumin (Durbin et al., 2017), resulting in increased apparent hydrodynamic radius (Frka-Petesic et al., 2016). Considering that BBB permeability declines exponentially with molecular size (Li et al., 2016), an increase in apparent hydrodynamic radius could impair payload delivery to brain tumors across the intact BBB or partially functional BTB. As demonstrated in our treatment study using the intracranial JIMT-1-BR3 tumor model (i.e., complete remission in five of six animals by homogeneous ADC **6** versus no complete remission by heterogeneous ADC **7**), a decrease in the effective DAR by heterogeneous conjugation could be further prominent in grade 1–3 gliomas and HER2-positive brain metastatic tumors, in which BBB disruption is less significant than in GBM (Gril et al., 2020; Yonemori et al., 2010). The use of more hydrophobic payloads than MMAF may also make this effect salient. As observed in previous studies using other solid tumor models (Hamblett et al., 2004; Lhospice et al., 2015), clearance and *in vivo* stability of ADCs could also be factors influencing payload delivery efficiency and overall treatment efficacy in orthotopic brain tumor models. Indeed, we observed promoted clearance for DAR 8 MMAF ADC **14**. However, DAR 6 ADC **13**, which also showed poor brain tumor targeting, did not undergo rapid clearance. In addition, the treatment efficacy of heterogeneous Cys conjugate **2** in the orthotopic U87ΔEGFR-luc tumor model was comparable with that of Lys conjugate **3**, despite its impaired thermal and circulation stability. Although heterogeneous ADCs with reduced average DARs may circumvent poor BBB penetration and brain tumor targeting caused by high-DAR components, such low-loading ADCs highly likely entail attenuated treatment efficacy. Overall, these findings support the following conclusions: (1) ADC homogeneity can influence payload delivery to brain tumors across the BBB more significantly than clearance and *in vivo* stability profiles, and (2) homogeneous ADC preparation at optimal DARs provides the best balance of brain-tumor-targeting capacity and payload delivery efficiency. Future in-depth structural and mechanistic studies will clarify the validity of our hypothesis in other combinations of mAbs, linker and conjugation chemistries, and payload types.

In summary, our findings highlight the critical importance of ADC homogeneity in maximizing efficacy in brain tumor treatment. Employing homogeneous conjugation at optimal DARs with properly designed linkers could be a promising approach to resurrecting the ADCs for GBM that have failed to show therapeutic benefits in clinical trials, including Depatux-M. Many payloads other than MMAF (e.g., monomethyl auristatin E [MMAE]) require a traceless release from their linker on internalization to exert full potency. When such ADC payloads are used, incorporating a proper cleavable linker will also be essential to ensure high therapeutic efficacy. In addition to this updated molecular design guideline, further understanding of brain tumor biology and pathophysiology will also be crucial to identify promising combinations of antibody targets, ADC linker properties (e.g., structure, drug release mechanism), and payload types. In particular, a deeper understanding of the integrity and functions of the BBB found in patient-derived brain tumor samples could open up the next step to improving payload delivery efficiency. Intratumor heterogeneity observed in many patients with gliomas is another critical issue making most targeted therapies ineffective; a part of glioma cells often lack target antigen expression, leading to tumor recurrence derived from non-responder cells (Inda et al., 2014; Parker et al., 2015; Shergalis et al., 2018). This issue could be overcome by using bispecific mAbs as a parent ADC scaffold, payloads with bystander killing effect (e.g., MMAE), or dual-drug ADCs developed by us recently (Yamazaki et al., 2021). Combination therapy with radiation and/or temozolomide may also substantially improve the efficacy of homogeneous ADCs in highly heterogeneous gliomas. We believe that such multifaceted approaches will finally lead us to promising ADCs or other targeted therapy modalities with the potential to conquer GBM and other intractable brain tumors.

## Limitations of the study

Several limitations should be noted for this study. First, most biodistribution studies were performed with a limited number of animals (n = 3–4/group). Although many previous studies have been performed with similar sample sizes, validation with a larger sample size would reveal the degree of impact by homogeneous conjugation on brain tumor targeting more accurately. Second, we used only one payload (MMAF) in this study. Optimal ADC design (e.g., linker type, DAR) likely varies when other payloads are used. Thus, structural optimization must be conducted for each combination of mAb, linker chemistry, and payload types. Lastly, the conclusion was drawn from studies using mouse models only. The cellular composition and tightness of the BBB have been shown to differ between murine and human brains (Jacobs et al., 2011). Thus, confirmatory studies should be performed using *in vitro* brain tumor models constituting human BBB (e.g., 3D organoid; Piantino et al., 2021) and patient samples of brain tumors before developing any clinical application based on our findings.

## STAR★METHODS

### RESOURCE AVAILABILITY

#### Lead contact

Further information and requests for resources and reagents should be directed to and will be fulfilled by the lead contact, Kyoji Tsuchikama (Kyoji.Tsuchikama@uth.tmc.edu).

#### Materials availability

All unique compounds and antibody conjugates generated in this study are available from the lead contact. There are no restrictions to the availability of these materials.

#### Data and code availability

- Synthetic schemes and characterization data of fluorescently labeled conjugates are reported in Data S1.
- This study did not generate large datasets, but raw data/images are available from the lead contact upon request.
- This paper does not report original codes.
- Any additional information required to reanalyze the data reported in this paper is available from the lead contact upon request.

### EXPERIMENTAL MODEL AND SUBJECT DETAILS

#### Cell lines

U87ΔEGFR was received from Dr. Erwin G. Van Meir (Emory University). Gli36δEGFR was received from Dr. E. Antonio Chiocca (Brigham and Women’s Hospital, Harvard Medical School). U87ΔEGFR-luc was generated by lentiviral transduction of U87ΔEGFR cells using Lentifect™ lentiviral particles encoding for firefly luciferase and a puromycin-resistant gene (GeneCopoeia, LP461-025). Transduction was performed according to the manufacturer’s instruction. U87ΔEGFR, U87ΔEGFR-luc, Gli36δEGFR, and HEK293 (ATCC) cells were cultured in DMEM (Corning) supplemented with 10% EquaFETAL® (Atlas Biologicals), GlutaMAX® (2 mM, Gibco), and penicillin-streptomycin (penicillin: 100 units/mL; streptomycin: 100 μg/mL, Gibco). JIMT1-BR3 was received from Dr. Patricia S. Steeg (National Cancer Institute) (Palmieri et al., 2014) and maintained in RPMI1640 (Corning) supplemented with 10% EquaFETAL®, GlutaMAX® (2 mM), sodium pyruvate (1 mM, Corning), and penicillin-streptomycin (penicillin: 100 units/mL; streptomycin: 100 μg/mL). JIMT-1-BR3 is a brain-tropic subline of the parent breast cancer cell line JIMT-1 (ER−, PR−, HER2+, and MGMT+) established by three rounds of intracardiac injection, formation of experimental brain metastases, sterile harvest, and *ex vivo* culture (Palmieri et al., 2014). JIMT-1-BR3 keeps positive MGMT status, making this cell line insensitive to temozolomide treatment. GBM12 was received from Dr. Jann N. Sarkaria (Mayo Clinic). RFP-expressing GBM12 (GBM12-RFP) was generated by transduction with lentivirus (System Biosciences, LL110VA-1) according to the manufacturer’s instruction. GBM12 and GBM12-RFP cells were maintained in DMEM supplemented with 2% fetal bovine serum and penicillin-streptomycin (penicillin: 100 units/mL; streptomycin: 100 μg/mL). All cells except U87ΔEGFR-luc and HEK293 were authenticated via short tandem repeat profiling before use. All cells were cultured at 37°C under 5% CO_2_, and passaged before becoming fully confluent up to 40 passages. All cells were periodically tested for mycoplasma contamination.

#### Animal studies

All procedures were approved by the Animal Welfare Committee of the University of Texas Health Science Center at Houston and performed in accordance with the institutional guidelines for animal care and use. All animals were housed under controlled conditions, namely 21–22°C (+/− 0.5°C), 30–75% (+/−10%) relative humidity, and 12:12 light/dark cycle with lights on at 7.00 a.m. Food and water were available ad libitum for all animals. NSG mice were purchased from The Jackson Laboratory (stock number: 005557) and bred in house. NSG mice (6–8 weeks old, male and female) were utilized in treatment studies for orthotopic xenograft mouse models of U87ΔEGFR-luc and GBM12 and *ex vivo* fluorescence imaging study. Male NSG mice (6–8 weeks old) were used in intravital imaging study. Female NSG mice (6–8 weeks old) were used in treatment study for orthotopic xenograft mouse models of JIMT1-BR3. CD-1® mice was purchased from Charles River Laboratories (Strain Code: 022) and used in *in vivo* pharmacokinetic study without in-house breeding. We did not perform power analysis prior to initiating each study. Instead, sample sizes were determined based on previous literature and/or our standard practices. Tumor-bearing mice were randomized so that all groups had similar starting tumor burdens. We used the following pre-defined humane endpoint in all animal studies: body weight loss of greater than 20% or signs of distress. The investigators were not blinded to allocation during experiments. No samples or animals were excluded from the studies.

### METHOD DETAILS

#### General information for chemistry

Unless otherwise noted, all materials for chemical synthesis were purchased from commercial suppliers (Acros Organics, AnaSpec, Broadpharm, Chem-Impex International, Fisher Scientific, Levena Biopharma, Sigma Aldrich, TCI America, and other vendors) and used as received. All anhydrous solvents were purchased and stored over activated molecular sieves under argon atmosphere.

Analytical reverse-phase high performance liquid chromatography (RP-HPLC) was performed using an Agilent LC-MS system consisting of a 1100 HPLC and a 1946D single quadrupole electrospray ionization (ESI) mass spectrometer equipped with a C18 reverse-phase column (Accucore™ C18 column, 3 × 50 mm, 2.6 μm, Thermo Scientific) or a Thermo LC-MS system consisting of a Vanquish UHPLC and a LTQ XL™ linear ion trap mass spectrometer equipped with a C18 reverse-phase column (Accucore™ Vanquish™ C18+ UHPLC column, 2.1 × 50 mm, 1.5 μm, Thermo Scientific). Standard analysis conditions for organic molecules were as follows: flow rate = 0.5 mL/min (for both systems); solvent A = water containing 0.1% formic acid; solvent B = acetonitrile containing 0.1% formic acid. Compounds were analyzed using a linear gradient and monitored with UV detection at 210 and 254 nm. Preparative HPLC was performed using a Breeze HPLC system (Waters) equipped with a C18 reverse-phase column (XBridge Peptide BEH C18 OBD Prep Column, 130Å, 5 μm, 19 × 150 mm, Waters). Standard purification conditions were as follows: flow rate = 20 mL/min; solvent A = water containing 0.05% trifluoroacetic acid (TFA), 0.1% formic acid or 0.1% NH_4_OH; solvent B = acetonitrile containing 0.05% TFA (standard conditions), 0.1% formic acid (FA conditions), or 0.1% NH_4_OH (basic conditions). Compounds were analyzed using a linear gradient and monitored with UV detection at 210 and 254 nm. In all cases, fractions were analyzed off-line using either of the LC-MS systems for purity confirmation and those containing a desired product were lyophilized using a Labconco Freezone 4.5 Liter Benchtop Freeze Dry System. High-resolution mass spectra were obtained using an Agilent 6530 Accurate Mass Q-TOF LC/MS or a Thermo Q Exactive™ Hybrid Quadrupole-Orbitrap™ Mass Spectrometer.

#### Synthesis of BCN-peg_3_-EVCit-PABC-MMAF (S2)

Fmoc-peg_3_-E(O*t*-Bu)VCit-PABC-MMAF (**S1**, 15 mg, 8.7 μmol, prepared as described previously (Yamazaki et al., 2021)) was dissolved in 50% diethylamine/DMF solution at room temperature. After 1 h, the solution was concentrated in vacuo and used in the next step without further purification. The crude products were dissolved in 50% TFA/DCM solution at room temperature. After being stirred at room temperature for 1 h, the solution was concentrated in vacuo and the crude compounds were precipitated with cold diethyl ether (5–6 mL) followed by centrifugation at 2,000 × *g* for 3 min (3 times). BCN-NHS(3.8 mg, 13.1 μmol, Berry&Associates) and DIPEA (7.6 μL, 43.5 μmol) were added to a solution of this crude mixture in DMF (1 mL) and the mixture was stirred at room temperature overnight. The crude products were purified by preparative RP-HPLC under basic conditions to afford analytically pure peptide **S2** (5.1 mg, 36% for the 3 steps). Purity was confirmed by LC-MS. White powder. HRMS (ESI) Calcd. For C_82_H_126_N_12_O_22_Na_2_ [M+2Na]^2+^: 838.4447, Found: 838.4467.

#### Synthesis of MMAF-NHS ester (S3)

DIPEA (42.5 μL, 0.24 mmol) was added to a solution of MMAF (102.9 mg, 0.12 mmol), di(*N*-succinimidyl) suberate (224.5 mg, 0.61 mmol), 1-hydroxy-7-azabenzotriazole (HOAt, 16.6 mg, 0.12 mmol) in DMF (2 mL) and the mixture was stirred at 37°C overnight. The crude products were purified by preparative RP-HPLC to afford analytically pure peptide **S3** (103.7 mg, 86%). Purity was confirmed by LC-MS. White powder. HRMS (ESI) Calcd. For C_51_H_80_N_6_O_13_Na [M + Na]^+^: 1007.5676, Found: 1007.5676.

#### Synthesis of Fmoc-peg_3_-E(O*t*-Bu)VCit-PABC-sarcosine (S4)

Bis(2,4-dinitrophenyl) carbonate (52.9 mg, 174 μmol) and DMAP (8.5 mg, 69.6 μmol) were added to a solution of Fmoc-peg_3_-E(O*t*-Bu) VCit-PABOH (34 mg, 34.8 μmol) in DMF (1 mL), and the mixture was stirred at room temperature for 2 h. To the mixture were added a solution of sarcosine (93 mg, 1.0 mmol) in water (1 mL) and additional DMF (0.5 mL) and the mixture was stirred at room temperature for 1 h. The crude products were purified by preparative RP-HPLC to afford analytically pure peptide **S4** (24.2 mg, 64%). Purity was confirmed by LC-MS. White powder. HRMS (ESI) Calcd. For C_54_H_74_N_8_O_16_Na [M + Na]^+^: 1113.5115, Found: 1113.5111.

#### Synthesis of Fmoc-peg_3_-EVCit-PABC-sarcosine NHS ester (S5)

Fmoc-peg_3_-E(O*t*-Bu)VCit-PABC-sarcosine (**S4**, 24.2 mg, 22.2 μmol), NHS (7.7 mg, 66.6 μmol), and EDC·HCl (12.8 mg, 66.6 μmol) were dissolved in DCM (1.5 mL) and the mixture was stirred at room temperature for 4 h. Then the reaction mixture was quenched with 15% citric acid and extracted with DCM. The organic layer was washed with brine, dried over Na_2_SO_4_, and concentrated. The crude products were dried in vacuo and used immediately in the next step without purification. The crude products were dissolved in 10% TFA/DCM solution. After being stirred at room temperature for 30 min, the solution was concentrated in vacuo and the residue was purified by preparative RP-HPLC to afford analytically pure peptide **S5** (12.4 mg, 49% for the 2 steps). Purity was confirmed by LC-MS. White powder. HRMS (ESI) Calcd. For C_54_H_70_N_9_O_18_ [M + H]^+^: 1132.4833, Found: 1132.4826.

#### Synthesis of H_2_N-peg_3_-EVCit-PABC-sarcosine-Cy5.5 (S6)

A solution of Cy5.5-amine (500 μL, 10 mM in DMSO, 5 μmol) and DIPEA (3.5 μL, 20 μmol) was added to a solution of Fmoc-peg_3_-EVCit-PABC-sarcosine NHS ester (**S5**, 500 μL, 10 mM in DMSO, 5 μmol) and the mixture was stirred at room temperature for 1.5 h. Additional NHS ester **S5** (200 μL, 10 mM in DMSO, 2 μmol) was added and the mixture was stirred at room temperature overnight. Diethylamine (800 μL) was added and the reaction mixture was stirred at room temperature for 1 h. The solution was concentrated in vacuo and the residue was purified by preparative RP-HPLC to afford analytically pure peptide **S6** (6.9 mg, 77% for the 2 steps). Purity was confirmed by LC-MS. Blue powder. HRMS (ESI) Calcd. For C_81_H_108_N_12_O_26_S_4_ [M−2H]^2−^: 896.3196, Found: 896.3188.

#### Synthesis of DBCO-peg_3_-EVCit-PABC-sarcosine-Cy5.5 (S7)

H_2_N-peg_3_-EVCit-PABC-sarcosine-Cy5.5 (**S6**, 6.2 mg, 3.45 μmol) was dissolved in DMF (500 μL) and DMSO (300 μL). To the solution were added DIPEA (1.2 μL, 6.9 μmol) and DBCO-NHS ester (1.8 mg, 4.49 μmol), and the mixture was stirred in the dark at room temperature overnight. The crude products were purified by preparative RP-HPLC under basic conditions to afford analytically pure peptide **S7** (6.5 mg, 90%). Purity was confirmed by LC-MS. Blue powder. HRMS (ESI) Calcd. For C_100_H_120_N_13_O_28_S_4_ [M−3H]^3−^: 692.9088, Found: 692.9085.

#### Synthesis of Boc-peg_4_-MMAF (S9)

Boc-peg_4_-acid (38.1 mg, 104 μmol), NHS (23.9 mg, 208 μmol), and EDC·HCl (39.7 mg, 208 μmol) were dissolved in DCM (0.75 mL) and the mixture was stirred at room temperature overnight. Then the reaction mixture was quenched with 15% citric acid and extracted with DCM. The organic layer was washed with brine, dried over Na_2_SO_4_, and concentrated. Crude Boc-peg_4_-NHS ester (**S8**) were dried in vacuo and used immediately in the next step without purification.

DIPEA (10 μL, 57.4 μmol) was added to a solution of MMAF (24.3 mg, 28.7 μmol), crude NHS ester **S8** (20 mg, 43 μmol, 100 mg/mL solution in DMSO), and HOAt (7.8 mg, 57.4 μmol) in DMF (200 μL) and the mixture was stirred at 37°C overnight. The crude products were purified by preparative RP-HPLC to afford analytically pure peptide **S9** (11.5 mg, 37%). Purity was confirmed by LC-MS. White powder. HRMS (ESI) Calcd. For C_55_H_95_N_6_O_15_ [M + H]^+^: 1079.6850, Found: 1079.6829.

#### Synthesis of BCN-peg_4_-MMAF (S10)

Boc-peg_4_-MMAF (**S9**, 11.5 mg, 10.7 mmol) was dissolved in 50% TFA/DCM solution. After being stirred at room temperature for 30 min, the solution was concentrated in vacuo and the crude compounds were precipitated with cold diethyl ether (10 mL) followed by centrifugation at 2,000 × *g* for 3 min (3 times). The residue was equally aliquoted into two vials for BCN and TCO installation, respectively.

For BCN installation, BCN-NHS (2.0 mg, 6.96 μmol, Berry&Associates) and DIPEA (0.9 μL, 10.7 μmol) were added to a solution of this crude mixture in DMF (300 μL) and the mixture was stirred at room temperature for 3 h. The crude products were purified by preparative RP-HPLC under FA conditions to afford analytically pure peptide S10 (2.8 mg, 45% for the 2 steps). Purity was confirmed by LC-MS. White powder. HRMS (ESI) Calcd. For C_61_H_99_N_6_O_15_ [M + H]^+^: 1155.7163, Found: 1155.7148. TCO-peg_4_-MMAF (**S11**) was synthesized in a similar manner.

#### Synthesis of TCO-peg_4_-MMAF (S11)

TCO-NHS (1.9 mg, 6.96 μmol) was used instead of BCN-NHS. 2.1 mg, 35% yield for the 2 steps. Purity was confirmed by LC-MS. White powder. HRMS (ESI) Calcd. For C_59_H_99_N_6_O_15_ [M + H]^+^: 1131.7163, Found: 1131.7151.

#### Antibodies

Anti-EGFR, anti-EGFRvIII, and anti-HER2 IgG1 mAbs with N88A/N297A, N297A, or N297Q mutation were expressed in-house (see below). The other antibodies used in this study were purchased from commercial vendors as follows: Rabbit anti-MMAF antibody (LEV-PAF1) from Levena Biopharma; goat anti-human IgG Fab-horseradish peroxidase (HRP) conjugate (109-035-097), goat anti-human IgG Fc antibody (109-005-098), and donkey anti-human IgG-HRP conjugate (709-035-149) from Jackson ImmunoResearch; goat anti-rabbit IgG–HRP conjugate (32260) from Thermo Fisher Scientific; rabbit anti-cleaved caspase 3 antibody (9661S) and rabbit anti-EGFR antibody (4267S) from Cell Signaling Technology); and rabbit anti-Ki67 antibody (ab16667) from Abcam.

#### Expression and purification of human monoclonal antibodies

All human monoclonal antibodies were produced according to the procedure reported previously (Anami et al., 2017; Shi et al., 2014). Briefly, free style HEK-293 human embryonic kidney cells (Invitrogen) were transfected with a mammalian expression vector encoding for the human IgG1 kappa light chain and full-length heavy chain sequences (based on variable sequences of cetuximab, depatuxizumab, ortrastuzumab). The transfected HEK-293 cells were cultured in a humidified cell culture incubator at 37°C with 8% CO_2_ and shaking at 150 rpm for 7 days before harvesting the culture medium. The antibody secreted into the culture medium was purified using Protein A resin (GE Healthcare).

#### MTGase-mediated antibody–linker conjugation

Anti-EGFR mAb with N88A/N297A double mutations (400 μL in PBS, 5.53 mg/mL, 2.21 mg antibody) was incubated with the diazide branched linker developed by us previously (Anami et al., 2017, 2018) (5.9 μL of 100 mM stock in water, 40 equiv.) and Activa TI® (101 μL of 40% solution in PBS, Ajinomoto, purchased from Modernist Pantry) at room temperature for 22 h. The reaction was monitored using either 1) an Agilent LC-MS system consisting of a 1100 HPLC and a 1946D single quadrupole ESI mass spectrometer equipped with a MabPac RP column (3 × 50 mm, 4 μm, Thermo Scientific) or 2) a Thermo LC-MS system consisting of a Vanquish UHPLC and a Q Exactive™ Hybrid Quadrupole-Orbitrap™ Mass Spectrometer equipped with a MabPac RP column (2.1 × 50 mm, 4 μm, Thermo Scientific). Elution conditions were as follows: mobile phase A = water (0.1% formic acid); mobile phase B = acetonitrile (0.1% formic acid); gradient over 6.8 min from A:B = 75:25 to 1:99; flow rate = 0.5 mL/min for the Agilent system or 0.25 mL/min for the Thermo system. The conjugated antibody was purified by SEC (Superdex 200 increase 10/300 GL, GE Healthcare, solvent: PBS, flow rate = 0.6 mL/min) to afford an antibody–linker conjugate (1.91 mg, 86% yield determined by bicinchoninic acid [BCA] assay).

#### Construction of homogeneous ADCs by strain-promoted azide–alkyne cycloaddition

BCN–EVCit–PABC–MMAF (20.7 μLof 3.7 mM stock solution in DMSO, 1.5 equivalent per azide group) was added to a solution of the mAb–linker conjugate in PBS (460 μL, 4.16 mg/mL), and the mixture was incubated at room temperature for 22 h. The reaction was monitored using either Agilent LC-MS system or Thermo LC-MS system equipped with a MabPac RP column (see above) and the crude products were purified by SEC to afford homogeneous ADC **1** (1.71 mg, 90% yield determined by BCA assay). Analysis and purification conditions were the same as described above. Homogeneity was confirmed by ESI-MS analysis. Homogeneous anti-EGFRvIII ADC **4** and anti-HER2 ADC **6** were prepared in the same manner.

#### Construction of a heterogeneous ADC by cysteine conjugation

Aglycosylated anti-EGFR mAb (298 μL in PBS, 3.0 mg/mL, 895 μg antibody) was mixed with TCEP (19.1 μL of 1 mM stock solution in water, 3.2 equiv.) and EDTA (30 μL of 10 mM stock solution in water, pH 8, 10% v/v) and incubated at 37°C for 2 h. MC–MMAF (9.0 μL of 10 mM stock solution in DMSO, 15 equiv.) was added to the partially reduced mAb solution and the reaction mixture was incubated overnight at room temperature. The reaction was monitored using an Agilent 1100 HPLC system equipped with a MAbPac HIC-Butyl column (4.6 × 100 mm, 5 μm, Thermo Scientific). Elution conditions were as follows: mobile phase A = 50 mM sodium phosphate containing ammonium sulfate (1.5 M) and 5% isopropanol (pH 7.4); mobile phase B = 50 mM sodium phosphate containing 20% isopropanol (pH 7.4); gradient over 25 min from A:B = 99:1 to 1:99; flow rate = 0.8 mL/min. *N*-acetyl cysteine (4.5 μL of 100 mM stock solution in DMSO, 75 equiv.) was added to the reaction mixture for quenching the reaction. The crude products were purified by SEC to afford Cys conjugate **2** (668 μg, 75% yield determined by BCA assay, average DAR: 3.8). SEC purification conditions were the same as described above. The average DAR value was determined based on UV peak areas in HIC analysis.

#### Construction of heterogeneous ADCs by lysine conjugation

Aglycosylated anti-EGFR mAb (105 μL in PBS, 3.0 mg/mL, 315 μg antibody) was mixed with 1 M phosphate solution at pH 9 (10.5 μL) and MMAF-NHS (2.5 μL of 10mM stock solution in DMSO, 12 equiv.) and the mixture was incubated at room temperature for 3 h. The reaction was monitored using either Agilent LC-MS system or Thermo LC-MS system equipped with a MabPac RP column (see above). The crude products were purified by SEC to afford Lys conjugate **3** (197 μg, 63% yield determined by BCA assay, average DAR: 3.9). Analysis and purification conditions were the same as described above. The average DAR value was determined based on ion intensity of each DAR species in ESI-MS analysis. Heterogeneous anti-EGFRvIII ADC **5** and anti-HER2 ADC **7** were constructed in the same manner.

#### Construction of anti-EGFR Cy5.5 conjugates

Cy5.5 conjugates **8–10** were prepared in the same manner as the preparation of corresponding ADCs described above. Instead of MMAF-containing linker modules, either of the following linker modules were used: DBCO–EVCit–Cy5.5 (synthesized in house, for homogeneous Cy5.5 conjugate **8**), Cy5.5 maleimide (purchased from Click Chemistry Tools, for Cys-Cy5.5 conjugate **9**), Cy5.5-NHS ester (purchased from Click Chemistry Tools, for Lys-Cy5.5 conjugate **10**), or DBCO–Cy5.5 (purchased from Click Chemistry Tools, for homogeneous non-cleavable Cy5.5 conjugate). Degrees of labeling (DOL) were determined by ESI-MS analysis (based on ion intensity of each DOL species) or using a plate reader (BioTek Synergy HTX) with a standard curve for free Cy5.5 (absorbance at 680 nm).

#### Construction of anti-EGFRvIII MMAF-Cy5.5 conjugates

Homogeneous anti-EGFRvIII MMAF ADCs with DARs of 4, 6, and 8 were prepared from depatuxizumab with an N297A (for DAR 4 and 6) or N297Q mutation (for DAR 8). For the preparation of the DAR 6 MMAF ADC, the diazido-methyltetrazine tri-arm linker developed by us previously (Yamazaki et al., 2021) was used. Subsequently, unmodified N297A depatuxizumab (DAR 0) and each ADC were labeled with Cy5.5-NHS ester (10 mM stock solution in DMSO, 6–8 equiv.) to achieve an average DOL of 2.3–2.5. The labeling reaction was performed in the same manner as described above, except that the reaction was quenched with ethanol amine (100 mM stock solution in water, 20 equiv.). The average DOL values of MMAF-Cy5.5 conjugates **11–14** were determined based on ion intensity of each DOL species in ESI-MS analysis.

#### Long-term stability test

Each ADC (1 mg/mL, 10 μL) in PBS was incubated at 37°C for 28 days and stored at −80°C until use. Samples were analyzed using an Agilent 1100 HPLC system equipped with a MAbPac SEC-1 analytical column (4.0 × 300 mm, 5 μm, Thermo Scientific). The conditions were as follows: flow rate = 0.2 mL/min; solvent = PBS. All assays were performed in triplicate.

#### Cell-based ELISA

Cells (U87ΔEGFR or HEK293) were seeded in a culture-treated 96-well clear plate (10,000 cells/well in 100 μL culture medium) and incubated at 37°C with 5% CO_2_ for 24 h. Paraformaldehyde (8%, 100 μL) was added to each well and incubated for 15 min at room temperature. The medium was discarded and the cells were washed three times with 100 μL of PBS. Cells were treated with 100 μL of blocking buffer (0.2% BSA in PBS) with agitation at room temperature for 2 h. After the blocking buffer was discarded, serially diluted samples (in 100 μL PBS containing 0.1% BSA) were added and the plate was incubated overnight at 4°C with agitation. The buffer was discarded and the cells were washed three times with 100 μL of PBS containing 0.25% Tween 20. Cells were then incubated with 100 μL of donkey anti-human IgG–HRP conjugate (diluted 1:10,000 in PBS containing 0.1% BSA) was added and the plate was incubated at room temperature for 1 h. The plate was washed three times with PBS containing 0.25% Tween 20, and 100 μL of 3,3′,5,5′-tetramethylbenzidine (TMB) substrate (0.1 mg/mL) in phosphate–citrate buffer/30% H_2_O_2_ (1:0.0003 volume to volume, pH 5) was added. After color was developed for 10–30 min, 25 μL of 3 N-HCl was added to each well and then the absorbance at 450 nm was recorded using a plate reader (BioTek Synergy HTX). K_D_ values were then calculated using Graph Pad Prism 8 software. All assays were performed in triplicate.

#### Cell viability assay

Cells were seeded in a culture-treated 96-well clear plate (5,000 cells/well in 50 μL culture medium) and incubated at 37°C under 5% CO_2_ for 24 h. Serially diluted samples (50 μL) were added to each well and the plate was incubated at 37°C for 72 h. After the old medium was replaced with 100 μL fresh medium, 20 μL of a mixture of WST-8 (1.5 mg/mL, Cayman chemical) and 1-methoxy-5-methylphenazinium methylsulfate (100 μM, Cayman Chemical) was added to each well, and the plate was incubated at 37°C for 2 h. After gently agitating the plate, the absorbance at 460 nm was recorded using a plate reader (BioTek Synergy HTX). EC_50_ values were calculated using Graph Pad Prism 8 software. All assays were performed in triplicate.

#### Orthotopic xenograft mouse models of human brain tumors

U87ΔEGFR-luc (1 × 10^5^ cells), GBM12 (2 × 10^5^ cells), or JIMT1-BR3 (2 × 10^5^ cells) were stereotactically implanted into NSG mice (6–8 weeks old, male and female) based on the previously reported method (Otani et al., 2020). Typical procedure. NSG mice were injected intraperitoneally with a cocktail of ketamine (67.5 mg/kg) and dexmedetomidine (0.45 mg/kg) and maintained at 37°C on a heating pad until the completion of surgery. After the head skin was shaved and treated with 10 μL of 0.25% bupivacaine supplemented with epinephrine (1:200,000), anesthetized mice were placed on a stereotactic instrument. After disinfecting the head skin with chlorhexidine and ethanol, a small incision was made and then a burr hose was drilled into the skull over the right hemisphere (1 mm anterior and 2 mm lateral to the bregma). A 10 μL Hamilton syringe (model 701 N) was loaded with cells suspended in 2 μL cold hanks-balanced salt solution (HBSS) and slowly inserted into the right hemisphere through the burr hole (3.5 mm depth). After a 1-min hold time, cells were injected over a 5-min period (0.4 μL/min). After a 3-min hold time, the needle was retracted at a rate of 0.75 mm/min. The incision was closed using GLUture® (Zoetis) and mice were injected with atipamezole (1 mg/kg, i.p.).

#### Treatment study

Brain tumor-bearing NSG mice were randomized and injected intravenously with a single dose of either ADC (3 mg/kg) or PBS. Group assignment and dose schedule were as follows: U87ΔEGFR-luc model, n = 4 or 6 for vehicle, n = 6 for ADCs, injected on Day 5; GBM12 model, n = 15 for vehicle, n = 14 for ADCs, injected on Day 8; JIMT-1-BR3 model, n = 6 for vehicle and homogeneous ADC **6**, n = 7 for heterogeneous ADC **8**, injected on Day 7. Growth of U87ΔEGFR-luc tumors was monitored by bioluminescence imaging (BLI) using an Xtreme *in vivo* imager (Bruker Biospin, upper limit: 1.5 × 10^5^ photons/sec/mm^2^; lower limit: 5.0 × 10^3^ photons/sec/mm^2^) once every week. The color contour and upper/lower limits of bioluminescence signals were adjusted for clear visualization without smear or high background noise. Tumor growth was also evaluated by MRI (see the following sections for details). Body weight was monitored every 3–4 days. Mice were euthanized when body weight loss of >20% or any severe clinical symptom was observed. Euthanasia of an animal was counted as a death.

#### MRI and measurement of tumor volume (GBM12 model)

MRI was performed using a 7 Tesla MRI scanner (Bruker Biospin) on Day 8 or 18 post tumor implantation. Tumor-bearing mice (n = 4/group, randomly selected from each group) were anesthetized with 1.5% isoflurane in a 30:70 mixture of O_2_ and medical air. MRI contrast agent (Dotarem) was injected (50 μL, i.p.) before imaging to help visualize the tumor. T2-weighted images were acquired using a multi-echo RARE sequence with a RARE factor of 3. Acquisition parameters were as follows: TR = 5000 ms, TE = 17 42.5 68 and 93.5 ms, 20–25 image slices with 500 μm slice thickness, in-plane resolution = 100 × 100 μm^2^. ImageJ software was utilized to measure the tumor volume. Regions of interest (ROI) were manually drawn to circumscribe the entire tumor, and volume was calculated by counting all the voxels within the ROI and multiplying the total number of pixels by the volume of the voxel (100 × 100 × 500 μm^3^).

#### MRI in the U87ΔEGFR-luc and JIMT-1-BR3 models

MRI images were taken using a 7 Tesla MRI scanner (Bruker Biospin) on Day 5 and 61 (U87ΔEGFR-luc) or Day 7 and 97 (JIMT-1-BR3) post tumor implantation. Tumor-bearing mice (U87ΔEGFR-luc model: 4 survivor mice treated with homogeneous anti-EGFRvIII ADC **4**; JIMT-1: n = 6/group) were anesthetized with 2% isoflurane throughout the imaging procedure. A 35 mm ID volume coil (Bruker Biospin) receive setup was used for data acquisition. T2-weighted coronal and axial images were acquired with a Spin Echo RARE sequence. Acquisition parameters were as follows: TR = 3000 ms, TE = 57 ms, RARE factor 12, 6 NAV, 20 slice images with thickness of 0.75 mm, slice gap 0.25 mm, in plane resolution of 156 μm for coronal and 117 μm for axial. Regions of interest (ROI) were manually drawn to circumscribe the entire tumor, and volume was calculated by counting all the voxels within the ROI and multiplying the total number of pixels by the volume of the voxel (156 × 117 × 750 μm^3^).

#### Immunohistochemistry

Mice were euthanized at the end of the treatment study in the GBM12 model and their excised tumor-bearing brain were embedded in paraffin. Samples were deparaffinized using xylene and rehydrated in decreasing concentration of ethanol. Subsequently, slices were incubated in 0.3% H_2_O_2_ for 30 min and autoclaved for 15 min at 121°C in citrate buffer. After blocking with animal-free blocking solution, slices were incubated with either rabbit anti-cCaspase 3 antibody (1:250), rabbit anti-EGFR antibody (1:50), or rabbit anti-ki67 antibody (1:200). SignalStain® Boost IHC Detection Reagent and DAB substrate kit (Cell Signaling Technology) were used and then the sections were counterstained with hematoxylin. Bright-field images were taken using an EVOS-FL Auto2 imaging system (Invitrogen). For cleaved caspase-3 and ki67 quantification, three representative areas of each stained sample were imaged and the populations of cCaspase3- and ki67-positive cells were analyzed using Image J software.

#### *In vivo* pharmacokinetic study

CD-1® mice (6–8 weeks old, female, n = 3/group) were injected intravenously with each mAb or ADC (3 mg/kg). Blood samples (5 μL) were collected from each mouse via the tail vein at each time point (15 min, 6 h, 1 day, 2 days, 4 days, 9 days, and 14 days) and immediately processed with 495 μL of 5 mM EDTA/PBS. After removal of cells by centrifugation (10 min at 10,000 × *g* at 4°C), plasma samples were stored at −80°C until use. All mice were humanely killed after last blood collection. Plasma samples were analyzed by sandwich ELISA. For determination of the total antibody concentration (both conjugated and unconjugated), a high-binding 96-well plate (Corning) was treated with goat anti-human IgG Fc antibody (500 ng/well). After overnight surface coating at 4°C, the plate was blocked with 100 μL of 2% BSA in PBS containing 0.05% Tween 20 (PBS-T) with agitation at room temperature for 1 h. Subsequently, the solution was removed and each diluted plasma sample (100 μL, diluted with PBS-T containing 1% BSA) was added to each well, and the plate was incubated at room temperature for 2 h. After each well was washed three times with 100 μL of PBS-T, 100 μL of goat anti-human IgG Fab–HRP conjugate (1:5,000) was added. After being incubated at room temperature for 1 h, the plate was washed and color development was performed as described above (see the section of “Cell-based ELISA”). For determination of ADC concentration (i.e., intact ADC-equivalent dose), assays were performed in the same manner using the following proteins and antibodies: human EGFR (100 ng/well, #EGR-H5222 from ACROBiosystems) for plate coating, and rabbit anti-MMAF antibody (1:5,000) and goat anti-rabbit IgG–HRP conjugate (1:10,000) as secondary and tertiary detection antibodies, respectively. Concentrations were calculated based on a standard curve generated using each intact ADC. As such, signal intensity declines in proportion to the loss of conjugated payloads, providing intact ADC-equivalent concentrations. Half-life at the elimination phase (t_1/2β_, day) and clearance rate [CL, (mg/kg)/(μg/mL)/day] of each conjugate were estimated using methods for non-compartmental analysis (Gabrielsson and Weiner, 2012). PKSolver (a freely available menu-driven add-in program for Microsoft Excel) (Zhang et al., 2010) was used for this calculation. Area under the curve (AUC_0–14 days_, μg/mL × day) was calculated using GraphPad Prism 8 software. See Table S3 for all observed PK parameters.

#### *Ex vivo* fluorescence imaging and quantification

Intracranial U87ΔEGFR-luc tumor-bearing NSG mice (6–8 weeks old, male and female) were prepared as described above and randomized into three groups (n = 3) 5 days post tumor implantation. Each Cy5.5 conjugate was administered intravenously at 3 mg/kg. After 48 h, the tumor-bearing mice were anesthetized with ketamine/xylazine. Subsequently, the mice underwent cardiac perfusion with PBS(+) containing sodium heparin (10 units/mL) and then 4% paraformaldehyde/PBS(+). This step removes conjugates circulating or bound to the vascular endothelial cells. Major organs including the brain were then harvested. Cy5.5-based near-infrared fluorescence images of the harvested organs were taken using a LI-COR Odyssey 9120 imager (Ex: 685 nm laser, intensity: L1.0 for brain, L2.0 for other organs, Em: 700 nm channel). Semi-quantification of the signals from ROIs was also performed using LI-COR Image Studio software. For tissue imaging, the brain samples were embedded in paraffin and tissue sections were prepared (thickness: 5 μm). After de-paraffinization of using toluene, mounting medium containing DAPI (VECTOR #H-1200) was applied to the tissue slides. Fluorescence Images were taken using a Nikon Eclipse TE2000E inverted microscope (Cy5 channel). Three ROIs in each sample were acquired and analyzed for semi-quantification using ImageJ software.

#### Intravital imaging

Male NSG mice (6–8 weeks old) were implanted with GBM12-RFP cells (2 × 10^5^ cells) stereotactically into the right hemisphere (2 mm lateral and 2.5 mm posterior to bregma, 1 mm depth) as previously described (Nair et al., 2020). Thirteen days after tumor implantation, craniectomy was performed over the tumor-implanted area. Cover glass (Bioscience Tools) was placed on the brain surface and glued to the skull with dental resin. Next day (Day 14), each Cy5.5 conjugate was administrated intravenously at 3 mg/kg (n = 4 for Cys-Cy5.5 conjugate **9**; n = 3 for the other groups). For intravital imaging, mice were anesthetized with isoflurane and positioned on the stage of a A1R-MP confocal microscope (NIKON) equipped with ×16 water immersion objective lens. Subsequently, 100 μL of 2% FITC-conjugated dextran (500 kDa, Sigma) was administrated through the tail vein, and Z-stack images were acquired based on Cy5.5, FITC, and RFP signals. Pre- and post-treatment images were acquired on Day 14, 15, 17, 19, and 22 after tumor implantation. The images were analyzed using NIS Elements AR software (NIKON). Intensity of the RFP (derived from GBM12-RFP) and Cy5.5 signals in two or three independent ROIs were calculated to determine Cy5.5/GBM12-RFP ratios.

### QUANTIFICATION AND STATISTICAL ANALYSIS

Curve-fitting analysis and statistical analysis were performed using GraphPad Prism 8 software. Kaplan-Meier survival curve statistics were analyzed with a log-rank (Mantel–Cox) test. To control the family-wise error rate in multiple comparisons, crude p values were adjusted by the Bonferroni method. Differences with adjusted p values < 0.05 were considered statistically significant in all analysis. For immunohistochemistry, immunofluorescence, *ex vivo* fluorescence imaging, MRI, and intravital imaging, a one-way ANOVA with a Tukey–Kramer or Dunnett’s post hoc test was used for multiple comparisons. See Table S4 and Figure legends for all p values and statistical test used.

## Supplementary Material

1

2

## Figures and Tables

**Table:** KEY RESOURCES TABLE

REAGENT or RESOURCE	SOURCE	IDENTIFIER
Antibodies		
N88A/N297A anti-EGFR mAb	This paper	N/A
N297A anti-EGFRvIII mAb	This paper	N/A
N297Q anti-EGFRvIII mAb	This paper	N/A
N297A anti-HER2 mAb	This paper	N/A
Rabbit anti-MMAF antibody	Levena Biopharma	Cat#LEV-PAF1
Goat anti-human IgG Fab-horseradish peroxidase (HRP) conjugate	Jackson ImmunoResearch	Cat#109-035-097; RRID:AB_2337585
Goat anti-human IgG Fc antibody	Jackson ImmunoResearch	Cat#109-005-098; RRID:AB_2337541
Donkey anti-human IgG-HRP conjugate	Jackson ImmunoResearch	Cat#709-035-149; RRID:AB_2340495
Goat anti-rabbit IgG–HRP conjugate	Thermo Fisher Scientific	Cat#32260; RRID:AB_1965959
Rabbit anti-cleaved caspase 3 antibody	Cell Signaling Technology	Cat#9661; RRID:AB_2341188
Rabbit anti-EGFR antibody	Cell Signaling Technology	Cat#4267; RRID:AB_2246311
Rabbit anti-Ki67 antibody	Abcam	Cat#Ab16667; RRID:AB_302459
Bacterial and virus strains		
HLUC-Lv105 Firefly Luciferase Lentifect™ Purified Lentiviral Particles	GeneCopoeia	Cat#LP461-025
pLL-CMV-RFP-T2A-Puro (Lenti-Labeler™ virus)	System Biosciences	Cat#LL110VA-1
Chemicals, peptides, and recombinant proteins		
Monomethyl auristatin F (MMAF)	Levena Biopharma	Cat# T1006
diazide branched linker	Anami et al., 2018	https://doi.org/10.1038/s41467-018-04982-3
BCN–EVCit–PABC–MMAF	This paper	N/A
MC–MMAF	This paper	CAS 863971-19-1
MMAF-NHS	This paper	N/A
DBCO–EVCit–Cy5.5	This paper	N/A
BCN-peg_4_-MMAF	This paper	N/A
TCO-peg_4_-MMAF	This paper	N/A
Click-easy™ BCN *N*-hydroxysuccinimide Ester	Berry & Associates	Cat#LK4320
TCO-NHS ester	BroadPharm	Cat#BP-22417
Sulfo-Cyanine5.5 amine (Cy5.5-amine)	Lumiprobe	Cat#273C0
Cy5.5 maleimide	Click Chemistry Tools	Cat#1113-1
Cy5.5-NHS ester	Click Chemistry Tools	Cat#1056-1
Activa TI®	Modernist Pantry	Cat#1002
Human EGFR Protein, His Tag (MALS verified)	ACROBiosystems	Cat#EGR-H5222
DAPI	VECTOR	Cat#H-1200
FITC-conjugated dextran	Sigma-Aldrich	Cat#FD500S
Homogeneous anti-EGFR ADC **1**	This paper	N/A
Cys conjugate **2**	This paper	N/A
Lys conjugate **3**	This paper	N/A
Homogeneous anti-EGFRvIII ADC **4**	This paper	N/A
Heterogeneous anti-EGFRvIII ADC **5**	This paper	N/A
Homogeneous anti-HER2 ADC **6**	This paper	N/A
Heterogeneous anti-HER2 ADC **7**	This paper	N/A
Homogeneous Cy5.5 conjugate **8**	This paper	N/A
Cys-Cy5.5 conjugate **9**	This paper	N/A
Lys-Cy5.5 conjugate **10**	This paper	N/A
Homogeneous Cy5.5 conjugate (non-cleavable)	This paper	N/A
DAR 0 mAb-Cy5.5 conjugate **11**	This paper	N/A
DAR 4 ADC-Cy5.5 conjugate **12**	This paper	N/A
DAR 6 ADC-Cy5.5 conjugate **13**	This paper	N/A
DAR 8 ADC-Cy5.5 conjugate **14**	This paper	N/A
Critical commercial assays		
BCA protein assay	Thermo Fisher Scientific	Cat#23225
WST-8	Cayman Chemical	Cat#18721
1-methoxy-5-methylphenazinium methylsulfate	Cayman Chemical	Cat#21258
SignalStain® Boost IHC Detection Reagent	Cell Signaling Technology	Cat#8114P
SignalStain® DAB Substrate Kit	Cell Signaling Technology	Cat#8059P
Experimental models: Cell lines		
U87ΔEGFR	Dr. Erwin G. Van Meir (Emory University)	N/A
U87ΔEGFR-luc	This paper	N/A
Gli36δEGFR	Dr. E. Antonio Chiocca (Brigham and Women’s Hospital, Harvard Medical School)	N/A
HEK293	ATCC	Cat#CRL-1573
JIMT1-BR3	Dr. Patricia S. Steeg (National Cancer Institute)	N/A
GBM12	Dr. Jann N. Sarkaria (Mayo Clinic)	N/A
RFP-expressing GBM12 (GBM12-RFP)	This paper	N/A
FreeStyle™ 293-F Cells	Thermo Fisher Scientific	Cat#R79007
Experimental models: Organisms/strains		
NOD.Cg-*Prkdc*^*scid*^ *Il2rg*^*tm1Wjl*^/SzJ mouse	The Jackson Laboratory	Cat#005557
CD-1® IGS Mouse	Charles River Laboratories	Cat#022
Software and algorithms		
GraphPad Prism 8	GraphPad Software	https://www.graphpad.com/scientific-software/prism/
Gen5	Agilent	https://www.biotek.com/products/software-robotics-software/gen5-microplate-reader-and-imager-software/
NIS Elements AR software	Nikon	https://www.microscope.healthcare.nikon.com/products/software/nis-elements
Microsoft Excel 16	Microsoft	https://www.microsoft.com/en-us/download/details.aspx?id=56547
Image J	NIH	https://imagej.nih.gov/ij/download.html
PKSolver version 2	Zhang et al., 2010	https://doi.org/10.1016/j.cmpb.2010.01.007
LI-COR Image Studio	LI-COR	https://www.licor.com/bio/image-studio/

## Highlights

- ADCs with optimal drug-to-antibody ratio show improved payload delivery to brain tumors
- Homogeneous ADCs show improved therapeutic effects in brain tumor models
- ADCs with high drug-to-antibody ratio poorly target brain tumors across the BBB
- ADC homogeneity is a critical parameter for maximizing efficacy in brain tumors
