# Supplementary material for: Homogeneity of antibody-drug conjugates critically impacts the therapeutic efficacy in brain tumors
Source: Cell Rep. Author manuscript; Available in PMC 2022 Jun 14. (PMC9195180; doi:10.1016/j.celrep.2022.110839)
Supplement: 1 [file NIHMS1810673-supplement-1.pdf]

Cell Reports, Volume *39*

Supplemental information

Homogeneity of antibody-drug conjugates critically impacts the therapeutic efficacy in brain tumors

Yasuaki Anami, Yoshihiro Otani, Wei Xiong, Summer Y.Y. Ha, Aiko Yamaguchi, Kimberly A. Rivera-Caraballo, Ningyan Zhang, Zhiqiang An, Balveen Kaur, and Kyoji Tsuchikama

Supplemental Information

Homogeneity of antibody-drug conjugates critically impacts the therapeutic efficacy in brain tumors

Yasuaki Anami[1], Yoshihiro Otani[2], Wei Xiong[1], Summer Y. Y. Ha[1], Aiko Yamaguchi[1], Kimberly A. Rivera-Caraballo[2], Ningyan Zhang[1], Zhiqiang An[1], Balveen Kaur[2], Kyoji Tsuchikama[1,3,*]

[1] Texas Therapeutics Institute, The Brown Foundation Institute of Molecular Medicine, McGovern Medical School, The University of Texas Health Science Center at Houston, Houston, Texas 77054, USA.

[2] Department of Neurosurgery, McGovern Medical School, The University of Texas Health Science Center at Houston, Houston, Texas 77030, USA.

[3]Lead Contact

*To whom correspondence should be addressed: Kyoji Tsuchikama (Kyoji.Tsuchikama@uth.tmc.edu)

Supplemental Figures

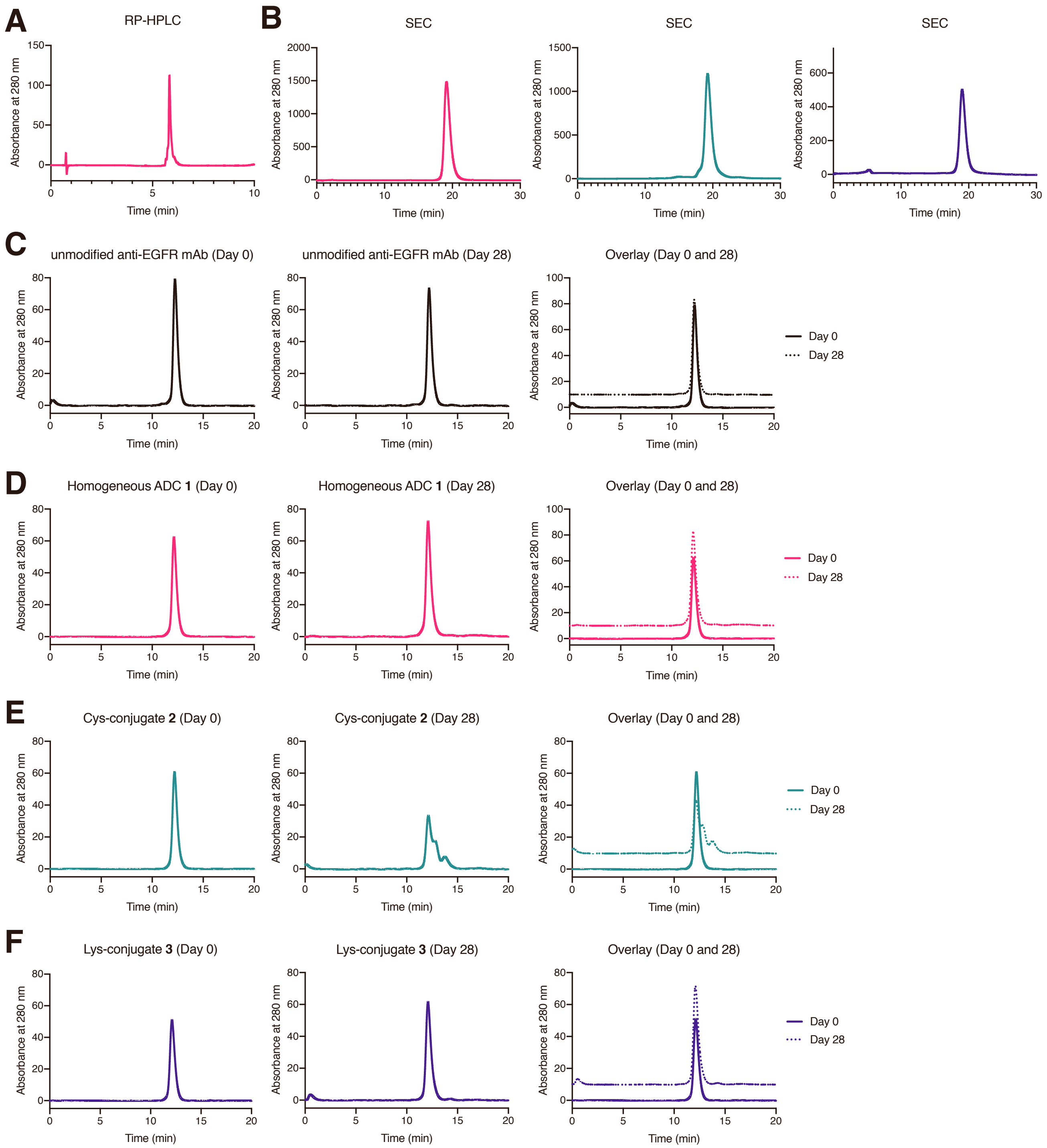

Figure S1. Reverse-phase HPLC and size-exclusion chromatography (SEC). Related to Figure 1. (A) Reverse-phase HPLC trace of homogeneous ADC **1** before SEC purification (UV absorbance: 280 nm). The average DAR was determined to be 4 based on the lack of lower DAR species. (B) SEC traces (UV absorbance: 280 nm) of homogeneous ADC **1** (magenta), Cys-conjugate **2** (green), and Lys-conjugate **3** (purple). (C–F) SEC analysis of ADCs after 1-month incubation at 37 ºC in PBS (pH 7.4). (C) Aglycosylated anti-EGFR mAb (cetuximab mutant), (D) homogeneous ADC **1**, (E) Cys-conjugate **2**, and (F) Lys-conjugate **3**.

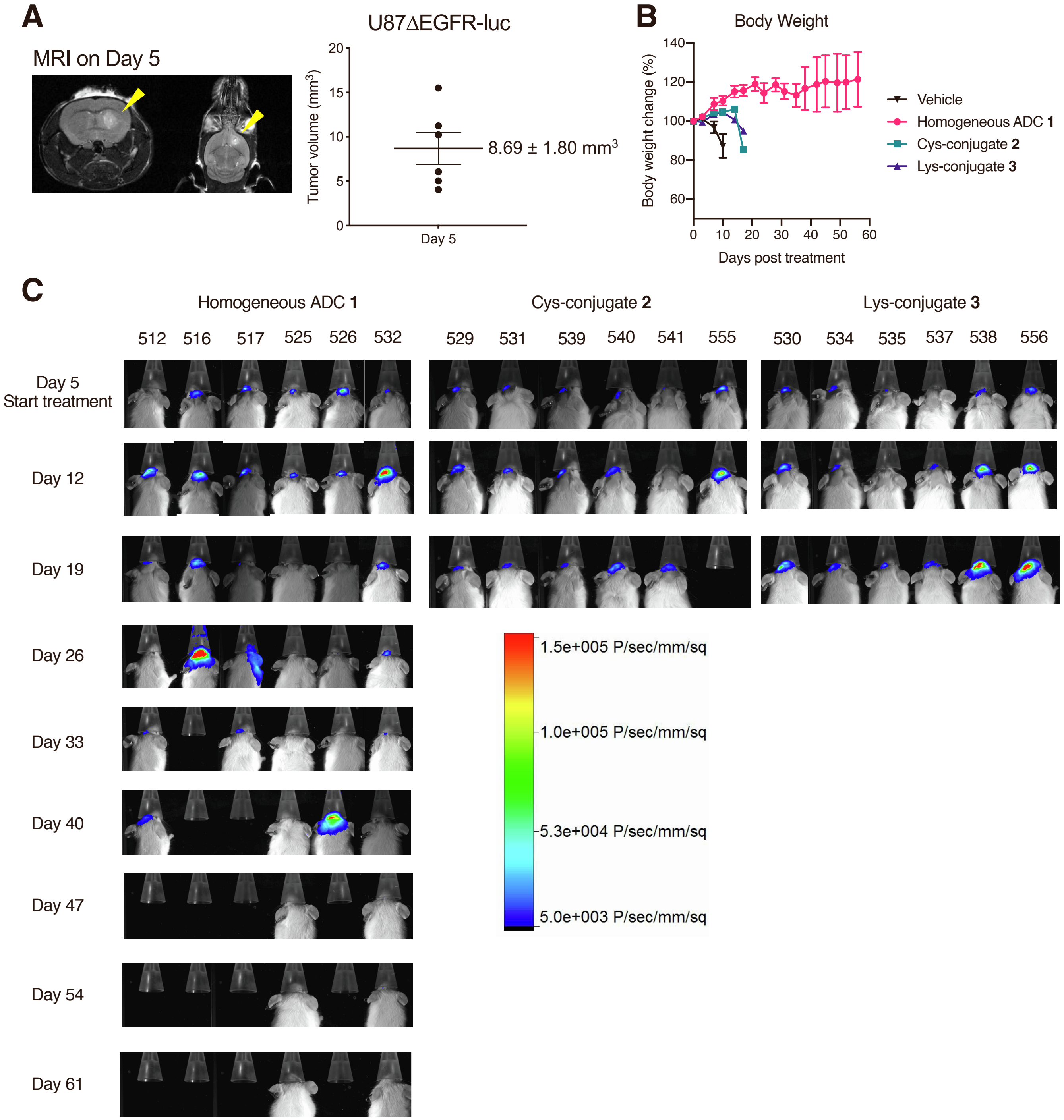

Figure S2. MRI, body weight change, and bioluminescence imaging in the orthotopic U87ΔEGFR-luc xenograft mouse model. Related to Figure 2. (A) Tumor volume measurement by MRI on Day 5. Initial tumor volume was 8.69 ± 1.80 mm^3 (n = 6). (B) Body weight change during treatment. Vehicle (black inversed triangle), homogeneous ADC **1** (magenta circle), Cys conjugate **2** (green square), and Lys conjugate **3** (purple triangle). Data are presented as mean values ± SEM (n = 6). (C) Bioluminescence images were taken right before ADC administration (Day 5) and then once a week. The color contour and upper/lower limits of bioluminescence signals were adjusted for clear visualization without smear or high background noise.

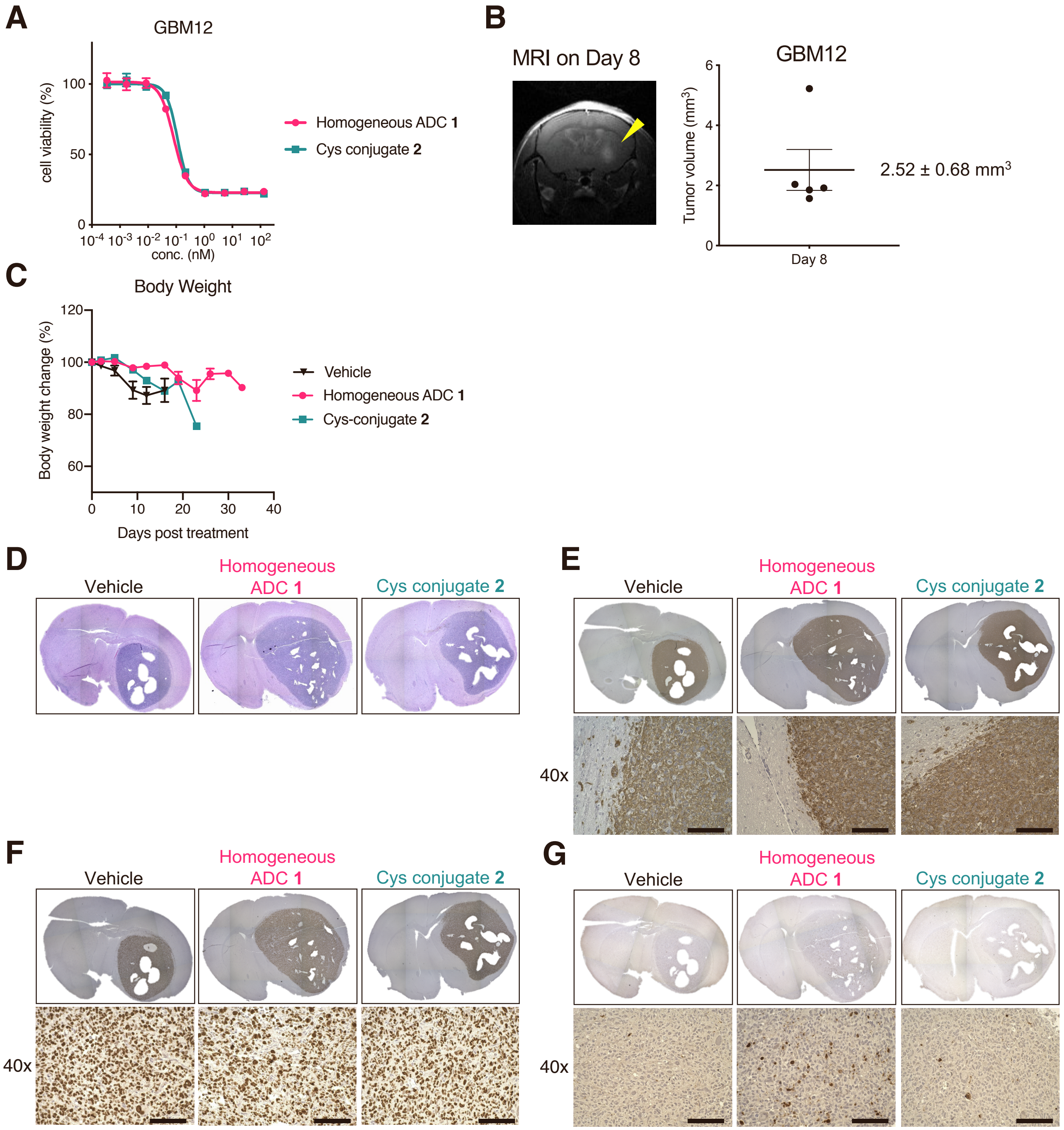

Figure S3. In vitro and in vivo evaluation of anti-EGFR ADCs in the GBM12 PDX model. Related to Figure 2. (A) In vitro cell killing potency in GBM12 cells. We tested homogeneous ADC **1** (magenta circle) and Cys conjugate **2** (green square). Concentrations are based on the antibody dose without normalizing to each DAR. All assays were performed in triplicate. (B) Tumor volume measurement by MRI on Day 8. Initial tumor volume was 2.52 ± 0.68 mm^3 (n = 5). (C) Body weight change during treatment in the orthotopic GBM12 xenograft mouse model. Vehicle (n = 15, black inversed triangle), homogeneous ADC **1** (n = 14, magenta circle), and Cys conjugate **2** (n = 14, green square). Data are presented as mean values ± SEM. (D–G) Immunohistochemistry analysis of GBM12 tumors harvested at the terminal stage. Representative images are shown. Tumor sections were stained with (D) H&E, (E) anti-human EGFR, (F) anti-Ki67, and (G) anti-cleaved-caspase 3 antibodies. Scale bar: 100 µm.

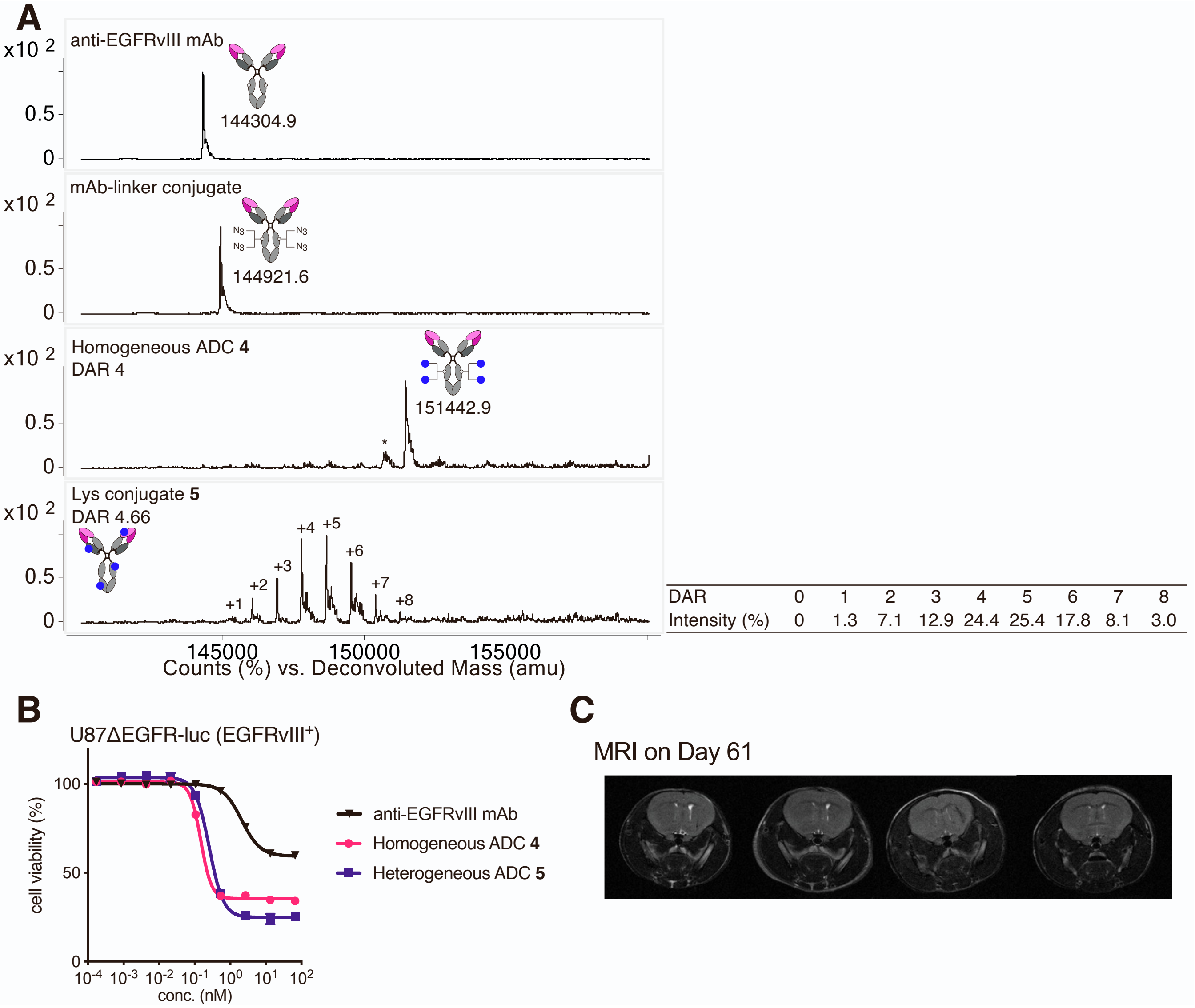

DAR	0	1	2	3	4	5	6	7	8
Intensity (%)	0	1.3	7.1	12.9	24.4	25.4	17.8	8.1	3.0

Figure S4. Characterization of anti-EGFRvIII ADCs and the anti-tumor effect in vitro and in vivo. Related to Figure 3. (A) Preparation and ESI-MS analysis of homogeneous anti-EGFRvIII ADC **4** and heterogeneous Lys conjugate **5**. First panel: N297A anti-EGFRvIII mAb (depatuxizumab mutant). Second panel: mAb–linker conjugate. Third panel: homogeneous ADC **4** with a DAR of 4. Asterisk (*) indicates a fragment ion detected in ESI-MS analysis. Fourth panel: Lys conjugate **5**. The average DAR was determined to be 4.66 based on the ion intensity of each DAR species. (B) In vitro cell killing potency in U87ΔEGFR-luc cells. We tested unmodified anti-EGFRvIII mAb (black inversed triangle), homogeneous ADC **4** (magenta circle) and heterogeneous ADC **5** (Lys conjugate **5**, purple square). Concentrations are based on the antibody dose without normalizing to each DAR. All assays were performed in triplicate. Data are presented as mean values ± SEM. (C) MRI analysis of 4 survivor mice that were intracranially implanted with U87ΔEGFR-luc cells and treated with a single dose of anti-EGFRvIII ADC **4** at 3 mg/kg on Day 8. The coronal images were taken on Day 61 post tumor implantation. No detectable tumor lesion was observed.

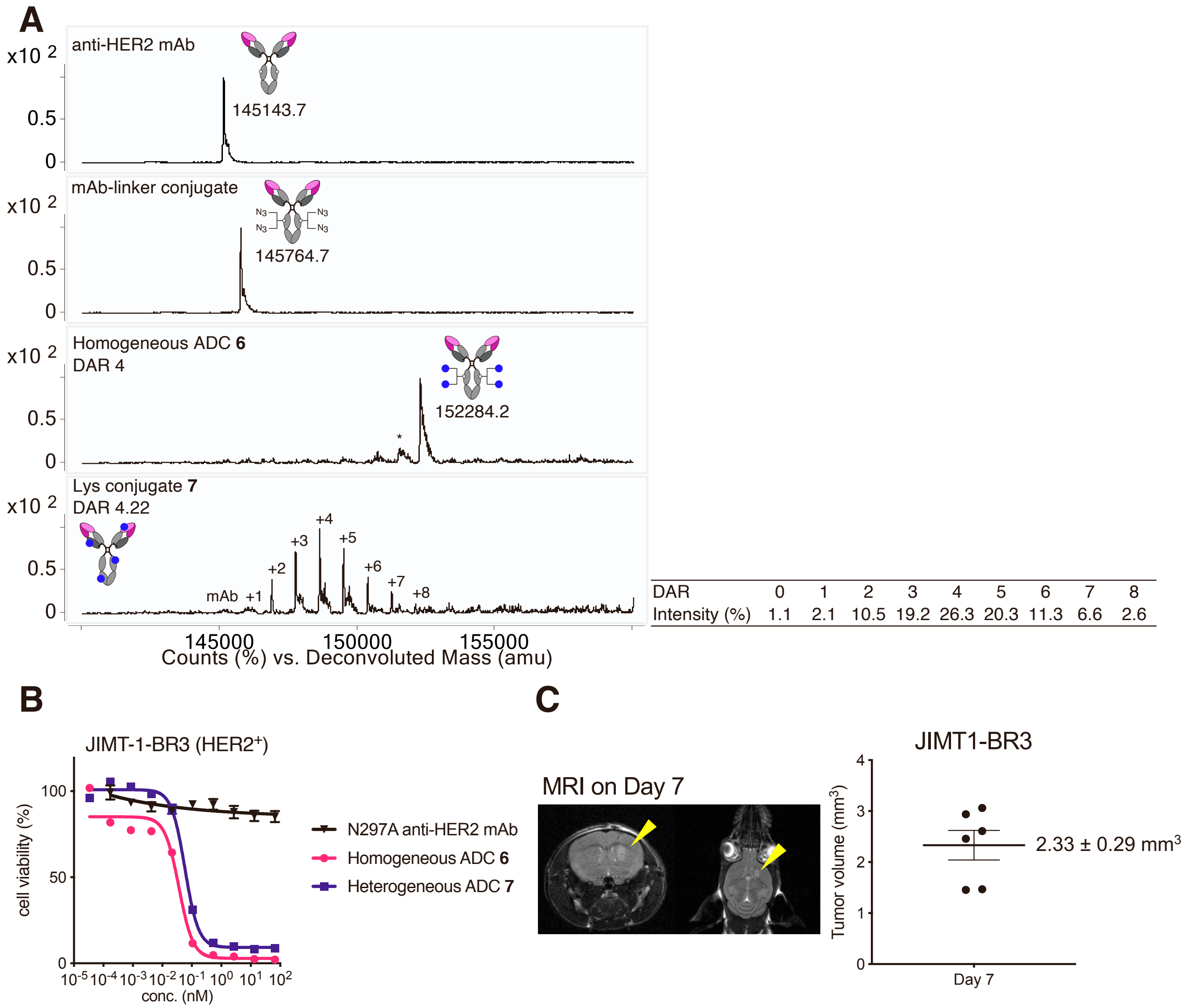

DAR	0	1	2	3	4	5	6	7	8
Intensity (%)	1.1	2.1	10.5	19.2	26.3	20.3	11.3	6.6	2.6

Figure S5. Characterization of anti-HER2 ADCs and the anti-tumor effect in vitro and in vivo. Related to Figure 3. (A) Preparation and ESI-MS analysis of homogeneous anti-HER2 ADC **6** and heterogeneous Lys conjugate **7**. First panel: N297A anti-HER2 mAb (trastuzumab mutant). Second panel: mAb–linker conjugate. Third panel: homogeneous ADC **6** with a DAR of 4. Asterisk (*) indicates a fragment ion detected in ESI-MS analysis. Fourth panel: Lys conjugate **7**. The average DAR was determined to be 4.22 based on the ion intensity of each DAR species. (B) In vitro cell killing potency in JIMT1-BR3 cells. We tested unmodified anti-HER2 mAb (black inversed triangle), homogeneous ADC **6** (magenta circle) and heterogeneous ADC **7** (Lys conjugate **7**, purple square). Concentrations are based on the antibody dose without normalizing to each DAR. All assays were performed in triplicate. Data are presented as mean values ± SEM. (C) Tumor volume measurement by MRI on Day 7. Initial tumor volume was 2.33 ± 0.29 mm^3 (n = 6).

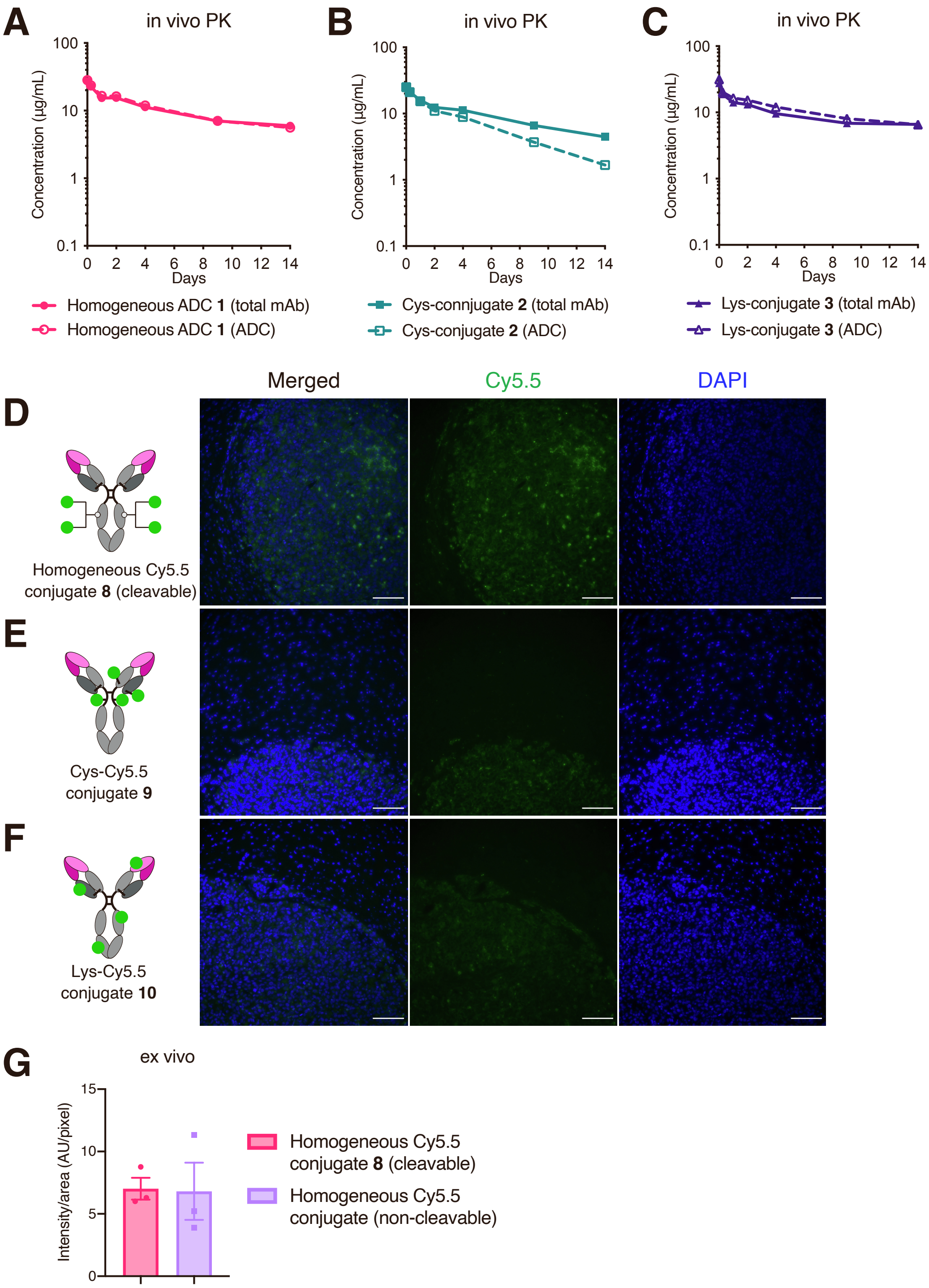

Figure S6. In vivo pharmacokinetics (PK) and biodistribution studies. Related to Figure 4. (A–C) Overlay PK curves of total antibody (conjugated and unconjugated, solid line) and ADC (conjugated only, dashed line) by sandwich ELISA. Data are presented as mean values ± SEM. (D–F) Tissue analysis of non-tumor and tumor region by fluorescent microscopy (20X magnification). Fluorescence images of brain tumor tissues harvested 48 hours after injecting each Cy5.5 conjugate (n = 3, scale bar: 100 μm). A representative image from each group is shown in all panels of fluorescence images. (G) Semi-quantification of the Cy5.5 signal detected in the whole orthotopic U87ΔEGFR-luc brain tumors treated with cleavable conjugate **8** or non-cleavable variant. Data are presented as mean values ± SEM.

Supplemental Tables

Table S1 K_D values of unmodified mAb and ADCs (n = 3). Related to Figure 1.

	K_D (nM)	CI95 (nM)
anti-EGFR mAb (cetuximab mutant)	0.039	0.036–0.043
Homogeneous ADC **1**	0.047	0.042–0.053
Cys conjugate **2**	0.044	0.040–0.048
Lys conjugate **3**	0.045	0.038–0.052

Calculated based on the data shown in Figure 1D.

Table S2 EC_{50} values of ADCs in GBM cell lines (n = 3). Related to Figure 1.

	EC_{50} (nM)	
	U87ΔEGFR	Gli36δEGFR
anti-EGFR mAb (cetuximab mutant)	1.99 (1.54–2.65)	–
Homogeneous ADC **1**	0.072 (0.064–0.083)	0.048 (0.044–0.052)
Cys conjugate **2**	0.110 (0.100–0.122)	0.035 (0.033–0.037)
Lys conjugate **3**	0.140 (0.126–0.156)	0.048 (0.044–0.053)

Calculated based on the data shown in Figure 1F and 1G. Values in parentheses are 95% confidential intervals.

Table S3 Summary of *in vivo* PK (n = 3). Related to Figure 4.

	$t_{1/2\beta\ \text{Total mAb}}$ (day)	$t_{1/2\beta\ \text{ADC}}$ (day)	$AUC_{0\text{-}14\ \text{Total mAb}}$ (μg/mL×day)	$AUC_{0\text{-}14\ \text{ADC}}$ (μg/mL×day)	$CL_{\text{obs Total mAb}}$ (mg/kg)/(μg/mL)/day	$CL_{\text{obs ADC}}$ (mg/kg)/(μg/mL)/day
anti-EGFR mAb (cetuximab mutant)	10.9	–	146.1 (127.5 – 164.7)	–	0.0119	–
Homogeneous ADC **1**	9.8	8.6	140.7 (129.2 – 152.1)	145.2 (135.0 – 155.4)	0.0126	0.0133
Cys conjugate **2**	7.8	4.2	129.6 (121.8 – 137.4)	97.2 (91.0 – 103.4)	0.0167	0.0279
Lys conjugate **3**	10.4	8.9	129.0 (117.5 – 140.5)	150.0 (141.2 – 158.9)	0.0137	0.0132

Calculated based on the data shown in Figure 4A and 4B. Values in parentheses are 95% confidential intervals. AUC, area under the curve; CL, clearance.

Table S4 Statistical significance. Related to Figures 2–6.

Main Figures	Method	Asterisk		Comparison	*P* value
Figure 2B	Log-rank[a] (Mantel-Cox)	**		Vehicle vs Homogeneous ADC **1**	$P = 0.0032$
		**		Vehicle vs Cys conjugate **2**	$P = 0.0032$
		**		Vehicle vs Lys conjugate **3**	$P = 0.0032$
		**		Homogeneous ADC **1** vs Cys conjugate **2**	$P = 0.0046$
		**		Homogeneous ADC **1** vs Lys conjugate **3**	$P = 0.0046$
		ns		Cys conjugate **2** vs Lys conjugate **3**	$P = 0.9000$
Figure 2D	Log-rank[a] (Mantel-Cox)	****		Vehicle vs Homogeneous ADC **1**	$P = 6.8 \times 10^{-8}$
		ns		Vehicle vs Cys conjugate **2**	$P = 0.12$
		****		Homogeneous ADC **1** vs Cys conjugate **2**	$P = 1.017 \times 10^{-5}$
Figure 2F	Tukey–Kramer	****		Vehicle vs Homogeneous ADC **1**	$P < 0.0001$
		**		Vehicle vs Cys conjugate **2**	$P = 0.0021$
		*		Homogeneous ADC **1** vs Cys conjugate **2**	$P = 0.0279$
Figure 2G	Tukey–Kramer	*		Vehicle vs Homogeneous ADC **1**	$P = 0.0149$
		*		Vehicle vs Cys conjugate **2**	$P = 0.0452$
		ns		Homogeneous ADC **1** vs Cys conjugate **2**	$P = 0.8010$
Figure 2H	Tukey–Kramer	**		Vehicle vs Homogeneous ADC **1**	$P = 0.0064$
		ns		Vehicle vs Cys conjugate **2**	$P = 0.6793$
		*		Homogeneous ADC **1** vs Cys conjugate **2**	$P = 0.0333$
Figure 3B	Log-rank (Mantel-Cox)	*		Homogeneous ADC **4** vs Lys conjugate **5**	$P = 0.0195$
Figure 3D	Log-rank (Mantel-Cox)	***		Homogeneous ADC **6** vs Lys conjugate **7**	$P = 0.0004$
Figure 4D	Tukey–Kramer	**		Homogeneous Cy5.5 conjugate **8** vs Cys-Cy5.5 conjugate **9**	$P = 0.0045$
		**		Homogeneous Cy5.5 conjugate **8** vs Lys-Cy5.5 conjugate **10**	$P = 0.0020$
		ns		Cys-Cy5.5 conjugate **9** vs Lys-Cy5.5 conjugate **10**	$P = 0.6589$
Figure 4F Kidney	Tukey–Kramer	*		Homogeneous Cy5.5 conjugate **8** vs Cys-Cy5.5 conjugate **9**	$P = 0.0144$
		ns		Homogeneous Cy5.5 conjugate **8** vs Lys-Cy5.5 conjugate **10**	$P = 0.9037$
		*		Cys-Cy5.5 conjugate **9** vs Lys-Cy5.5 conjugate **10**	$P = 0.0235$
Figure 4G Liver	Tukey–Kramer	*		Homogeneous Cy5.5 conjugate **8** vs Cys-Cy5.5 conjugate **9**	$P = 0.0357$
		ns		Homogeneous Cy5.5 conjugate **8** vs Lys-Cy5.5 conjugate **10**	$P = 0.8889$
		ns		Cys-Cy5.5 conjugate **9** vs Lys-Cy5.5 conjugate **10**	$P = 0.0632$
Figure 5E	Dunnett's test	*	Day +1	Homogeneous Cy5.5 conjugate **8** vs Cys-Cy5.5 conjugate **9**	$P = 0.0153$
		*	Day +1	Homogeneous Cy5.5 conjugate **8** vs Lys-Cy5.5 conjugate **10**	$P = 0.0267$
		*	Day +3	Homogeneous Cy5.5 conjugate **8** vs Cys-Cy5.5 conjugate **9**	$P = 0.0188$
		*	Day +3	Homogeneous Cy5.5 conjugate **8** vs Lys-Cy5.5 conjugate **10**	$P = 0.0202$
		*	Day +5	Homogeneous Cy5.5 conjugate **8** vs Cys-Cy5.5 conjugate **9**	$P = 0.0318$
		ns	Day +5	Homogeneous Cy5.5 conjugate **8** vs Lys-Cy5.5 conjugate **10**	$P = 0.3210$
Figure 6C	Dunnett's test	ns		DAR 4 ADC **12** vs DAR 0 mAb **11**	$P = 0.8483$
		*		DAR 4 ADC **12** vs DAR 6 ADC **13**	$P = 0.0396$
		*		DAR 4 ADC **12** vs DAR 8 ADC **14**	$P = 0.0288$
Figure 6E	Dunnett's test	***		DAR 4 ADC **12** vs DAR 0 mAb **11**	$P = 0.0006$
		ns		DAR 4 ADC **12** vs DAR 6 ADC **13**	$P = 0.2450$
		ns		DAR 4 ADC **12** vs DAR 8 ADC **14**	$P = 0.7237$
Figure 6F	Dunnett's test	****		DAR 4 ADC **12** vs DAR 0 mAb **11**	$P < 0.0001$
		ns		DAR 4 ADC **12** vs DAR 6 ADC **13**	$P = 0.0683$
		***		DAR 4 ADC **12** vs DAR 8 ADC **14**	$P = 0.0003$

[a] *P* values were adjusted by the Bonferroni correction for multiple comparisons. *$P < 0.05$; **$P < 0.01$; ***$P < 0.001$; ****$P < 0.0001$.
