# Supplementary material for: Homogeneity of antibody-drug conjugates critically impacts the therapeutic efficacy in brain tumors
Source: Cell Rep. Author manuscript; Available in PMC 2022 Jun 14. (PMC9195180; doi:10.1016/j.celrep.2022.110839)
Supplement: 2 [file NIHMS1810673-supplement-2.pdf]

Supplemental Data S1 Synthetic schemes and characterization of chemical compounds and Cy5.5 labeled antibody–drug conjugates. Related to the STAR Methods.

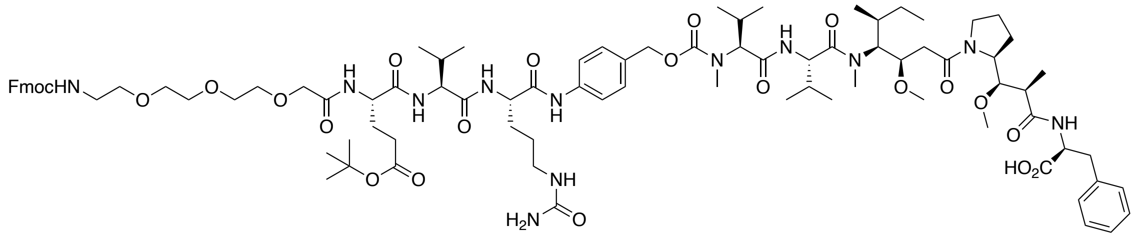

S1

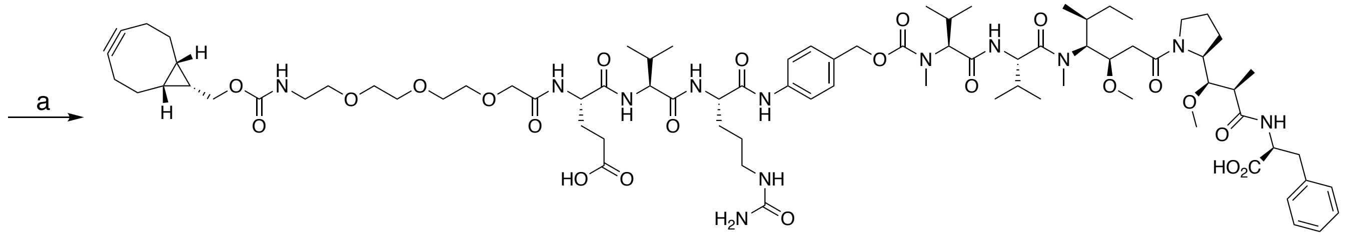

BCN-peg$_3$-EVCit-MMAF **S2** (36%, 3 steps)

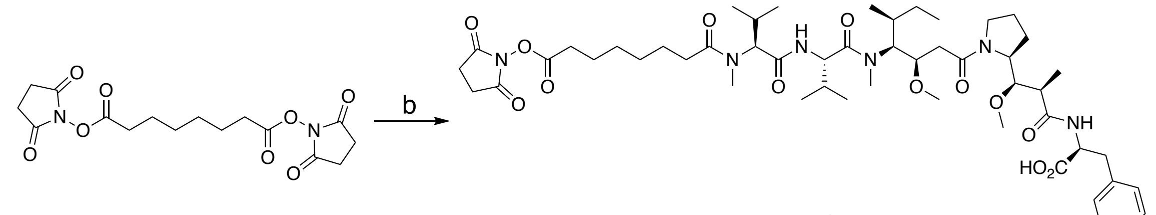

Di(*N*-succinimidyl) Suberate

MMAF-NHS **S3** (86%)

Synthesis of BCN-EVCit-MMAF module S2 and MMAF-NHS ester (S3). Reagents and conditions: (a) 50% diethylamine/DMF, rt, 1 h, and 50% TFA/DCM, rt, 1 h, then MMAF, DIPEA, HOAt, DMF, rt, overnight. (b) MMAF, DIPEA, HOAt, di(*N*-succinimidyl) suberate, 37 °C, overnight.

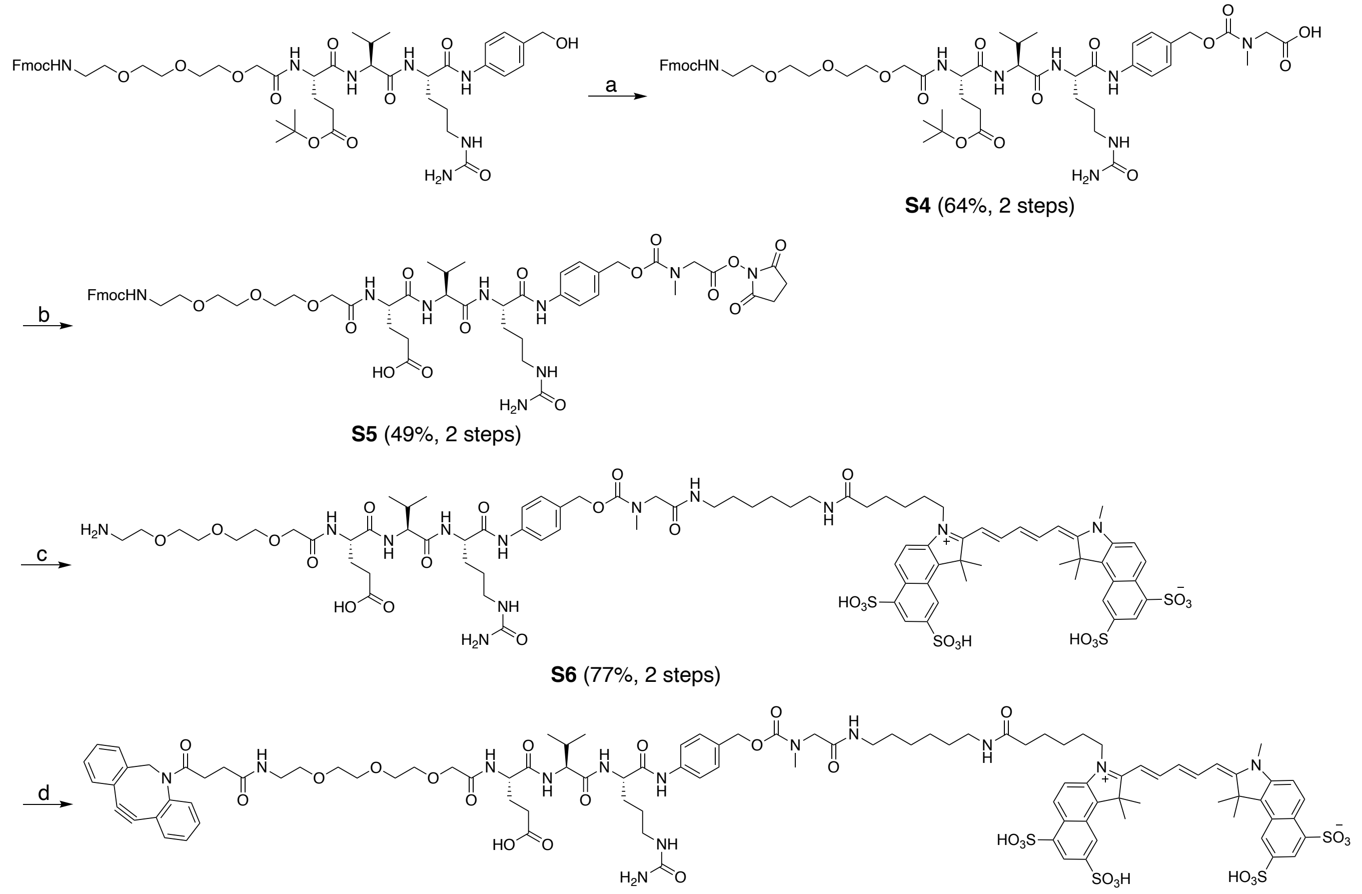

DBCO-peg$_3$-EVCit-Cy5.5 **S7** (90%)

Synthesis of DBCO-EVCit-Cy5.5 module S7. Reagents and conditions: (a) bisPNP, DMAP, DMF, rt, 2 h, then sarcosine, rt, 1h. (b) NHS, EDC·HCl, DCM, then 10% TFA/DCM. (c) Cy5.5-amine, DIPEA, rt, overnight, then diethylamine. (d) DBCO-NHS, DIPEA, DMF, DMSO, rt, overnight.

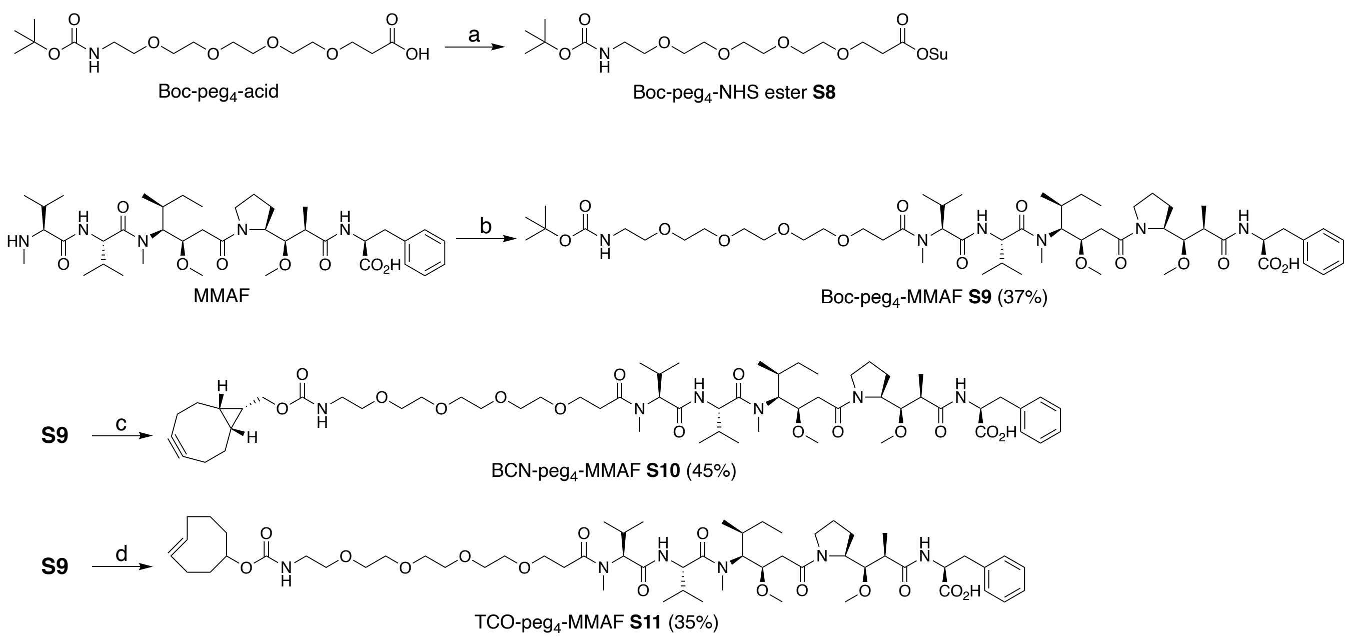

Synthesis of non-cleavable MMAF modules S10 and S11. Reagents and conditions: (a) NHS, EDC·HCl, DCM, rt, overnight. (b) **S8**, HOAt, DIPEA, DMF, 37 ºC, overnight. (c) 50% TFA/DCM, rt, 30 min, then BCN-NHS, DIPEA, DMF, rt, 3 h. (d) 50% TFA/DCM, rt, 30 min, then TCO-NHS, DIPEA, DMF, rt, 3 h.

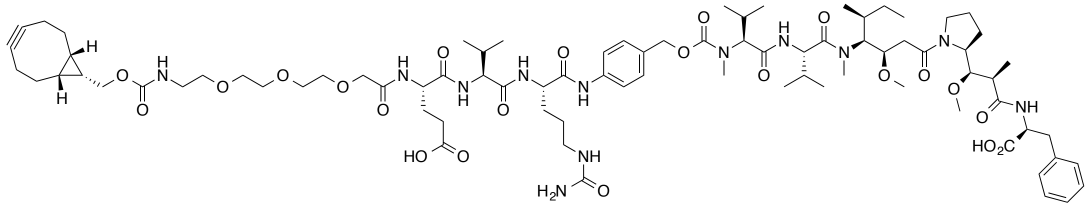

BCN-peg$_3$-EVCit-PABC-MMAF (**S2**)

Print of all graphic windows

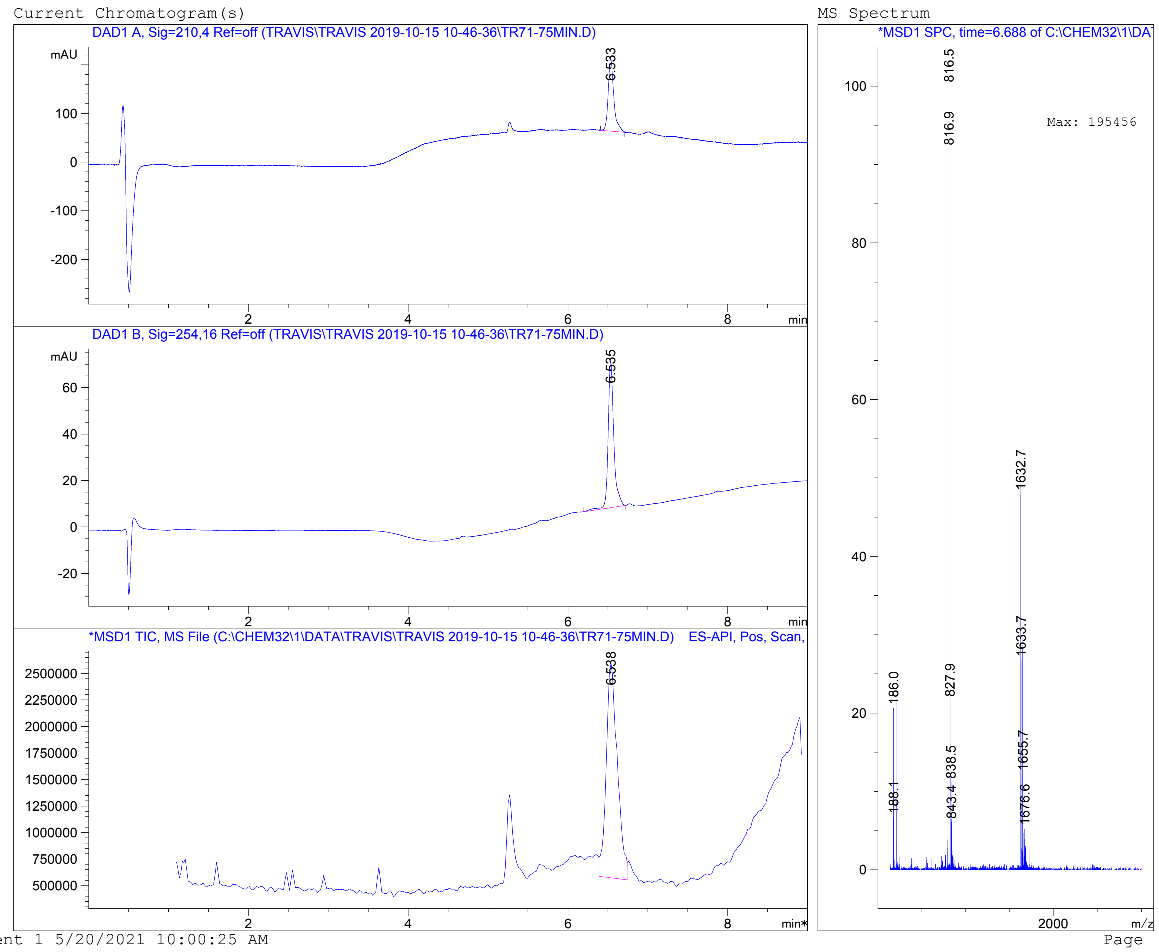

Instrument 1 5/20/2021 10:00:25 AM Page 1 of 1

HPLC trace and low-resolution ESI-MS spectrum of pure product S2.

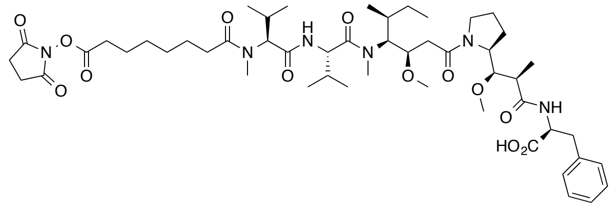

MMAF-NHS ester (**S3**)

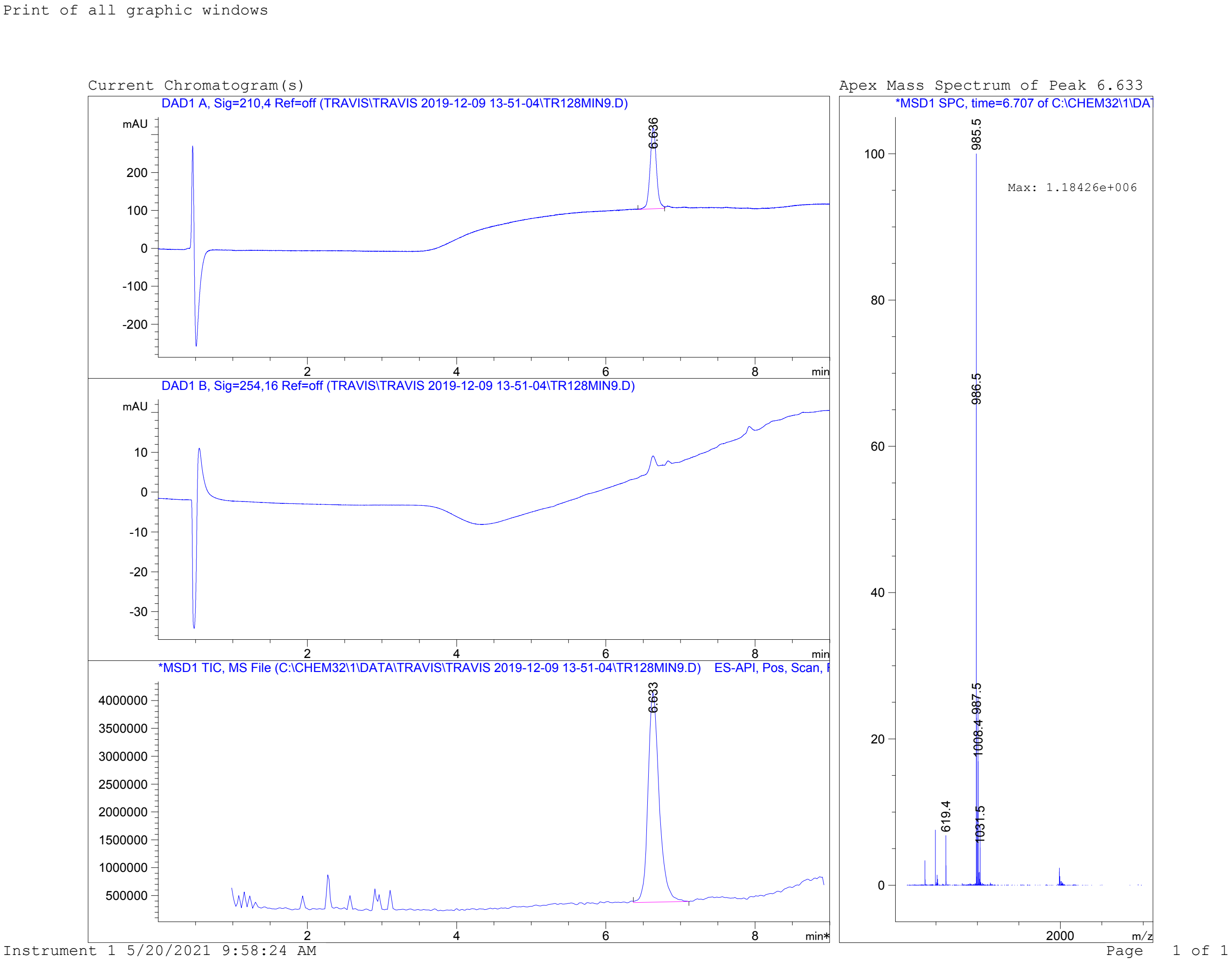

HPLC trace and low-resolution ESI-MS spectrum of pure product S3.

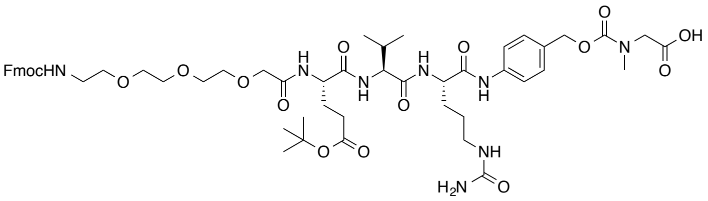

Fmoc-peg$_3$-E(O*t*-Bu)VCit-PABC-sarcosine (**S4**)

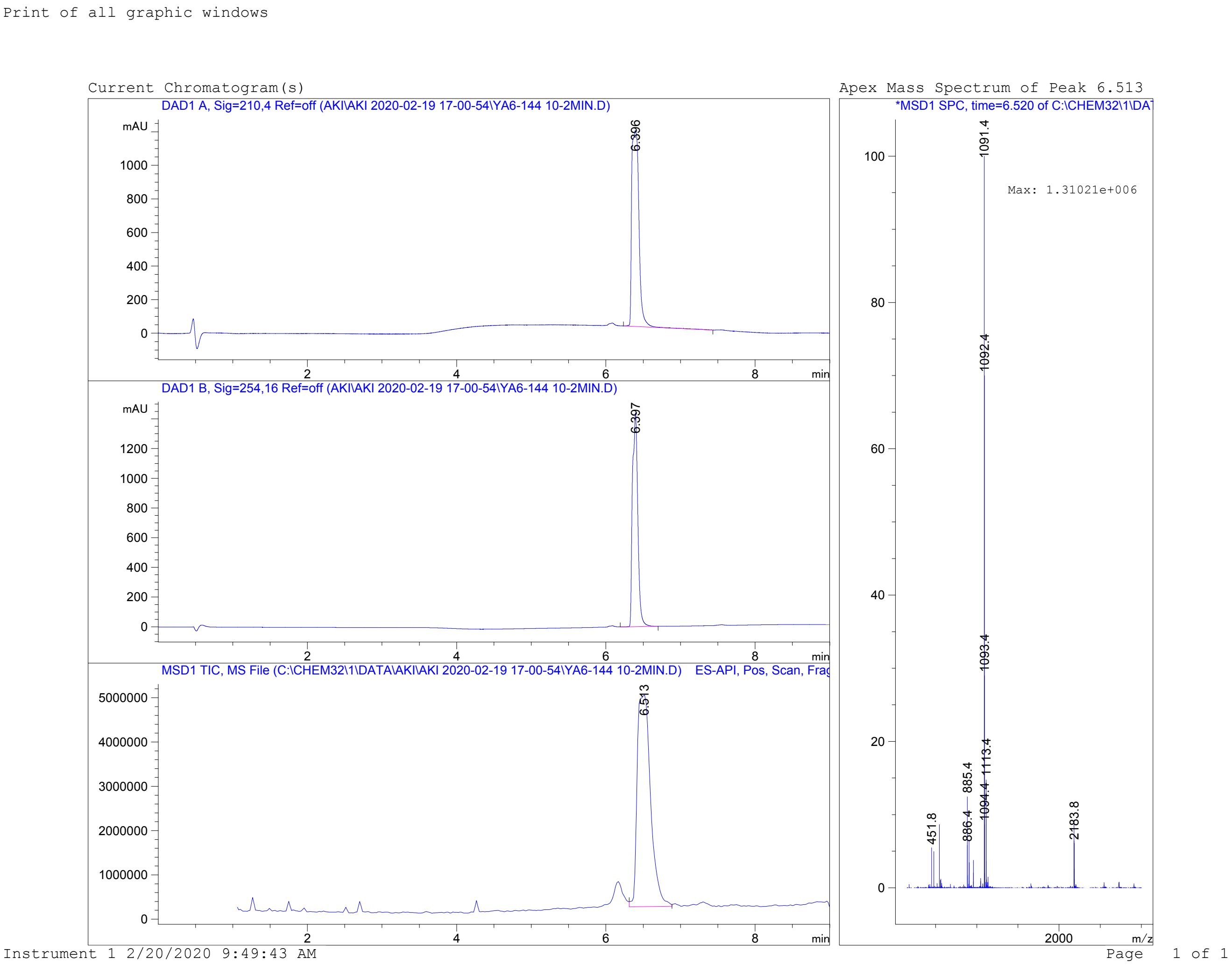

HPLC trace and low-resolution ESI-MS spectrum of pure product S4.

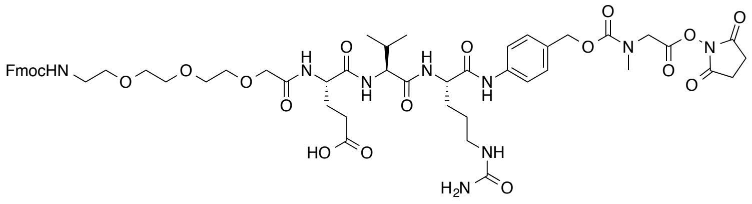

Fmoc-peg$_3$-EVCit-PABC-sarcosine NHS ester (**S5**)

Print of all graphic windows

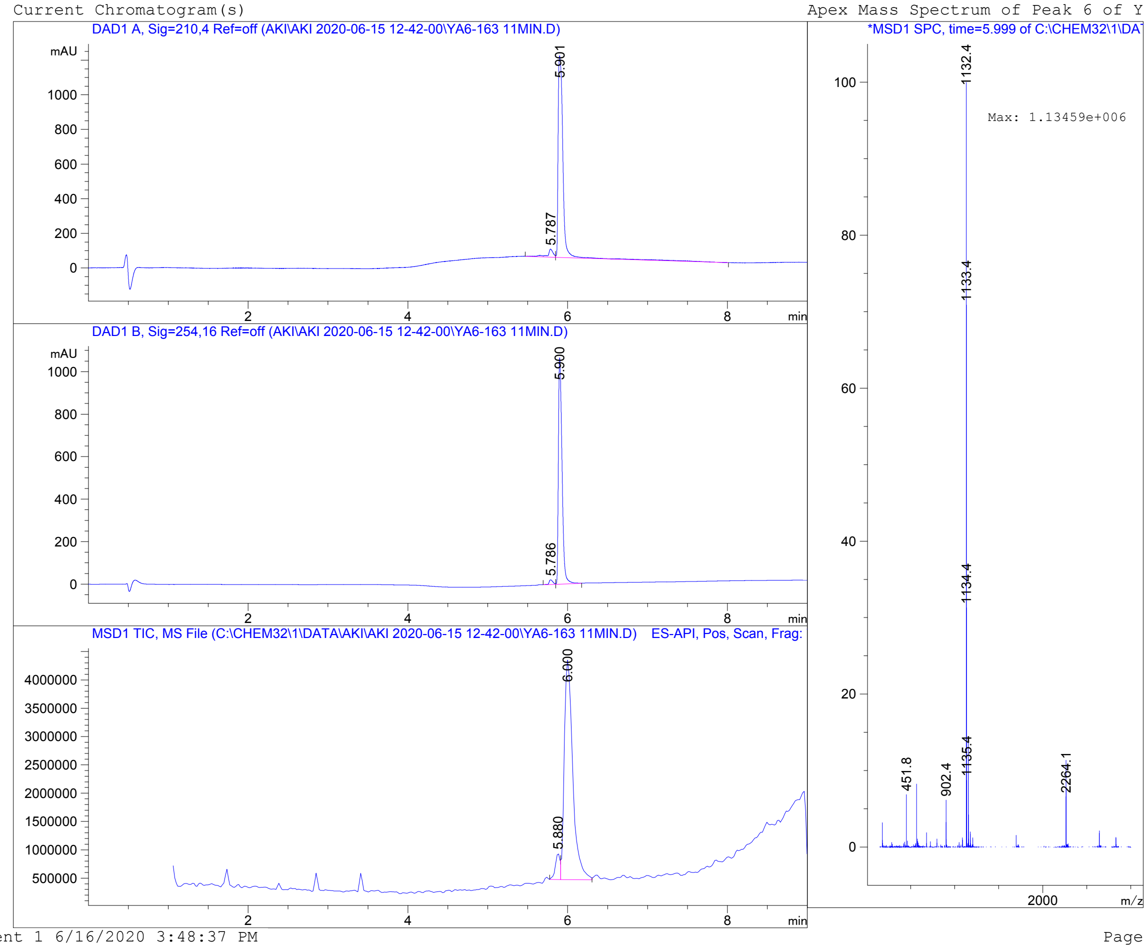

Instrument 1 6/16/2020 3:48:37 PM Page 1 of 1

HPLC trace and low-resolution ESI-MS spectrum of pure product S5.

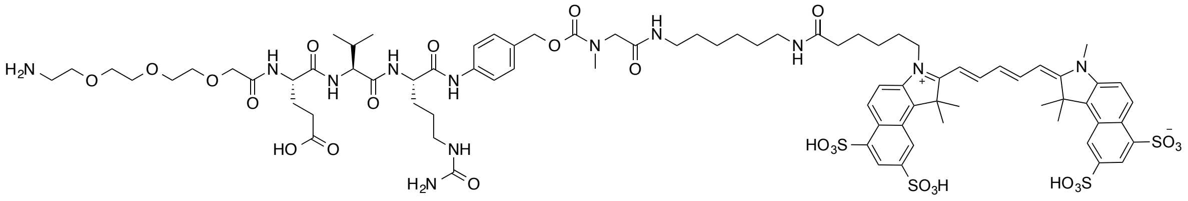

H_2N-peg$_3$-EVCit-PABC-sarcosine-Cy5.5 (**S6**)

Print of all graphic windows

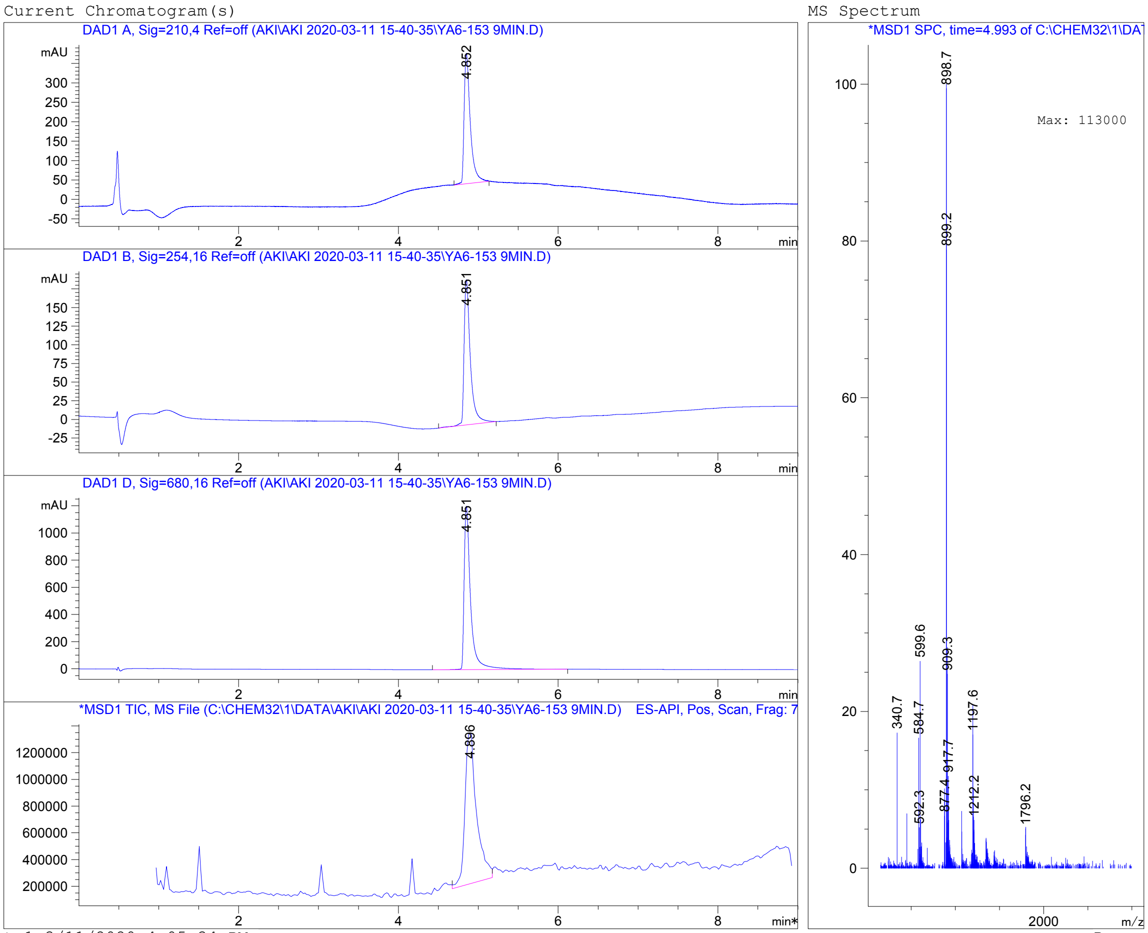

Instrument 1 3/11/2020 4:05:34 PM Page 1 of 1

HPLC trace and low-resolution ESI-MS spectrum of pure product S6.

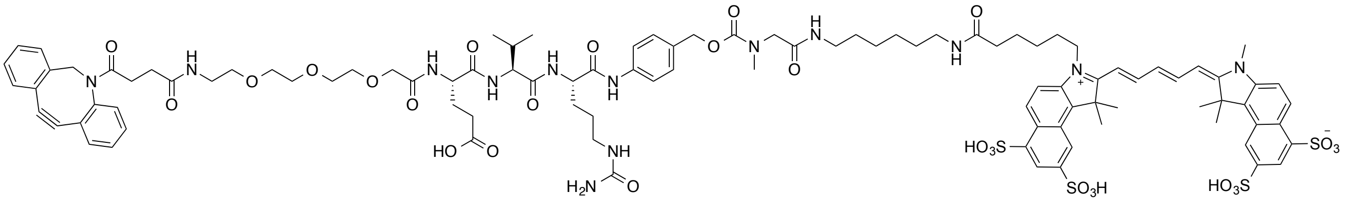

DBCO-peg$_3$-EVCit-PABC-sarcosine-Cy5.5 (**S7**)

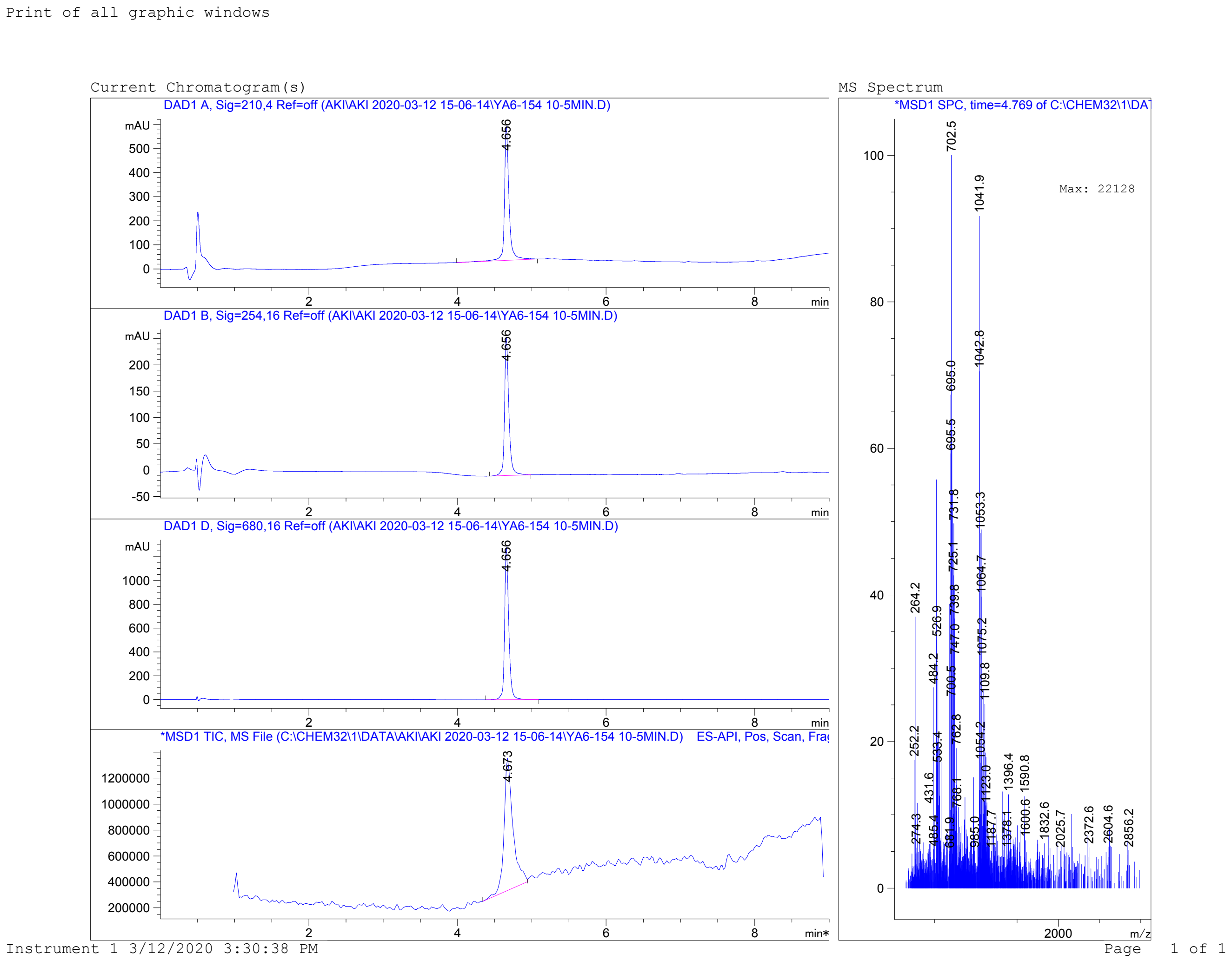

HPLC trace and low-resolution ESI-MS spectrum of pure product S7.

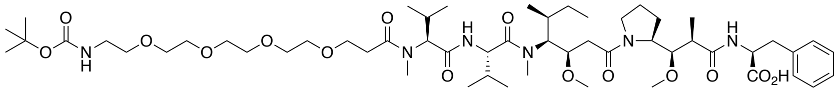

Boc-peg$_4$-MMAF (**S9**)

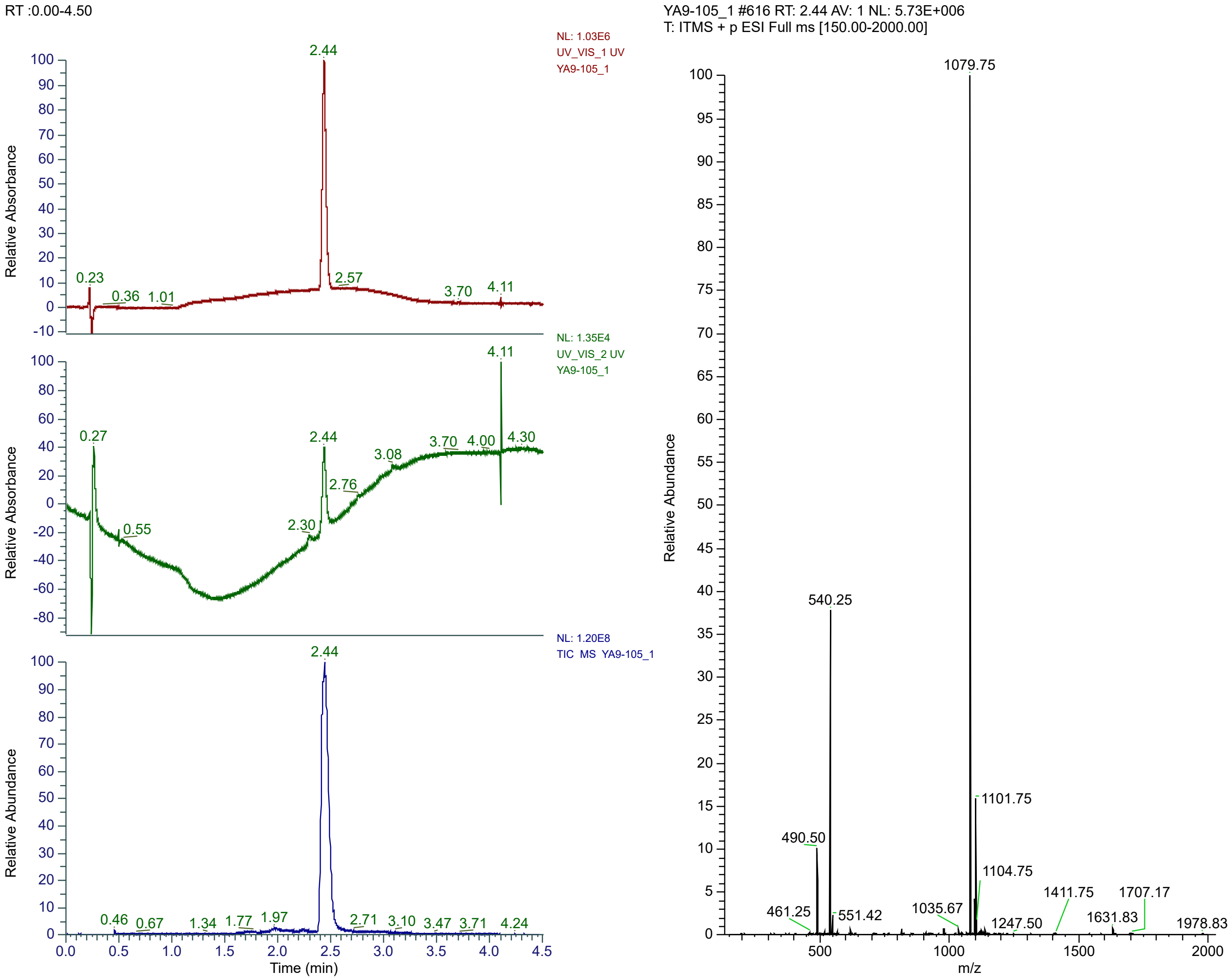

HPLC trace and low-resolution ESI-MS spectrum of pure product S9.

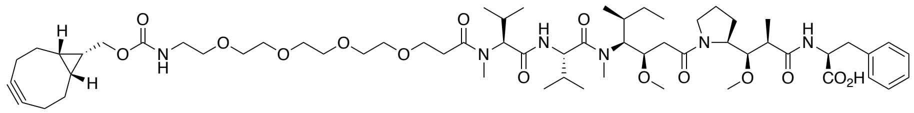

BCN-peg$_4$-MMAF (**S10**)

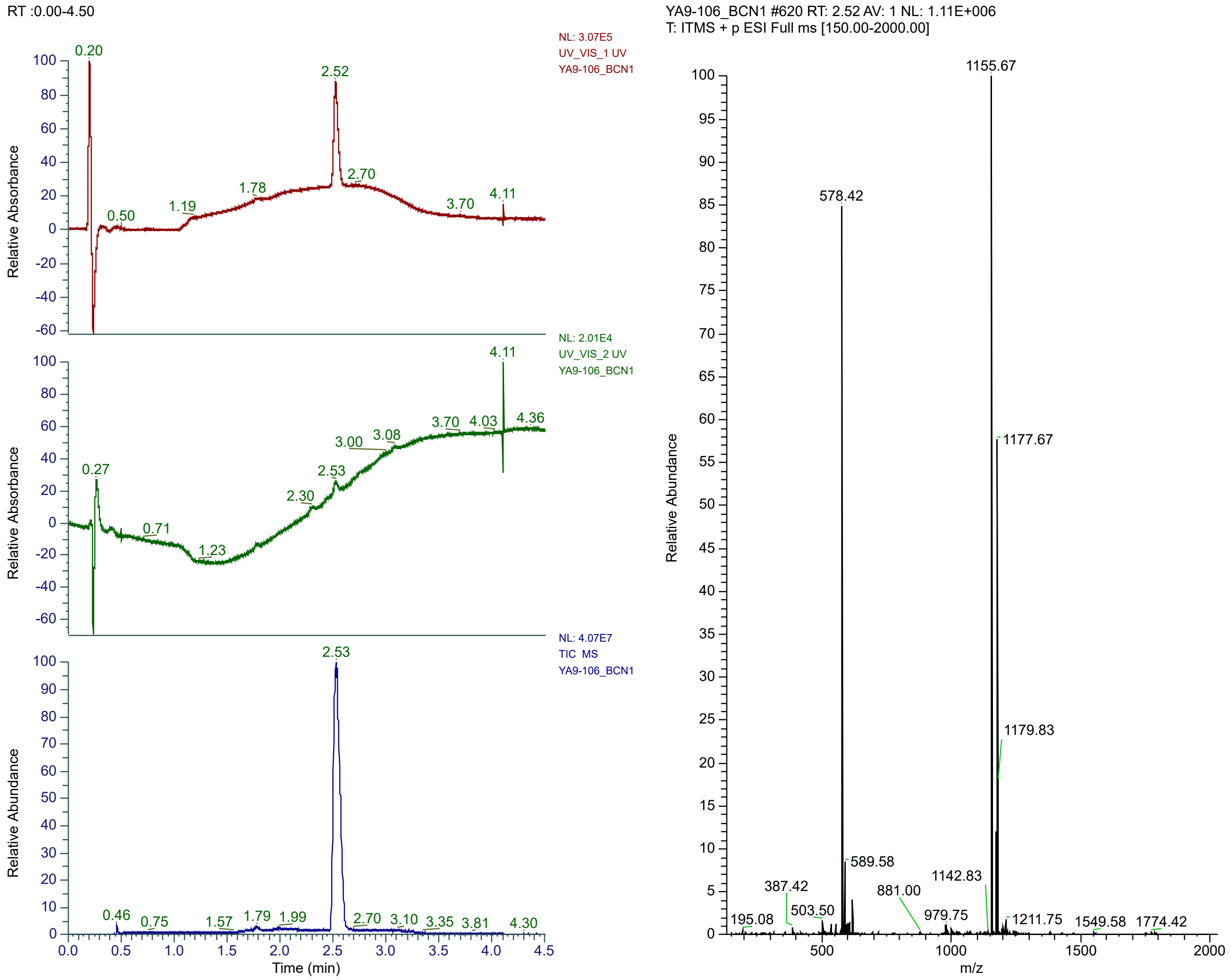

HPLC trace and low-resolution ESI-MS spectrum of pure product S10.

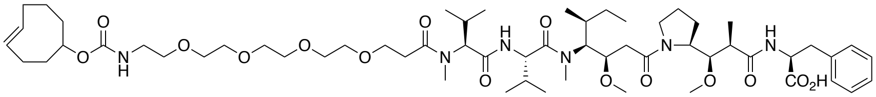

TCO-peg$_4$-MMAF (**S11**)

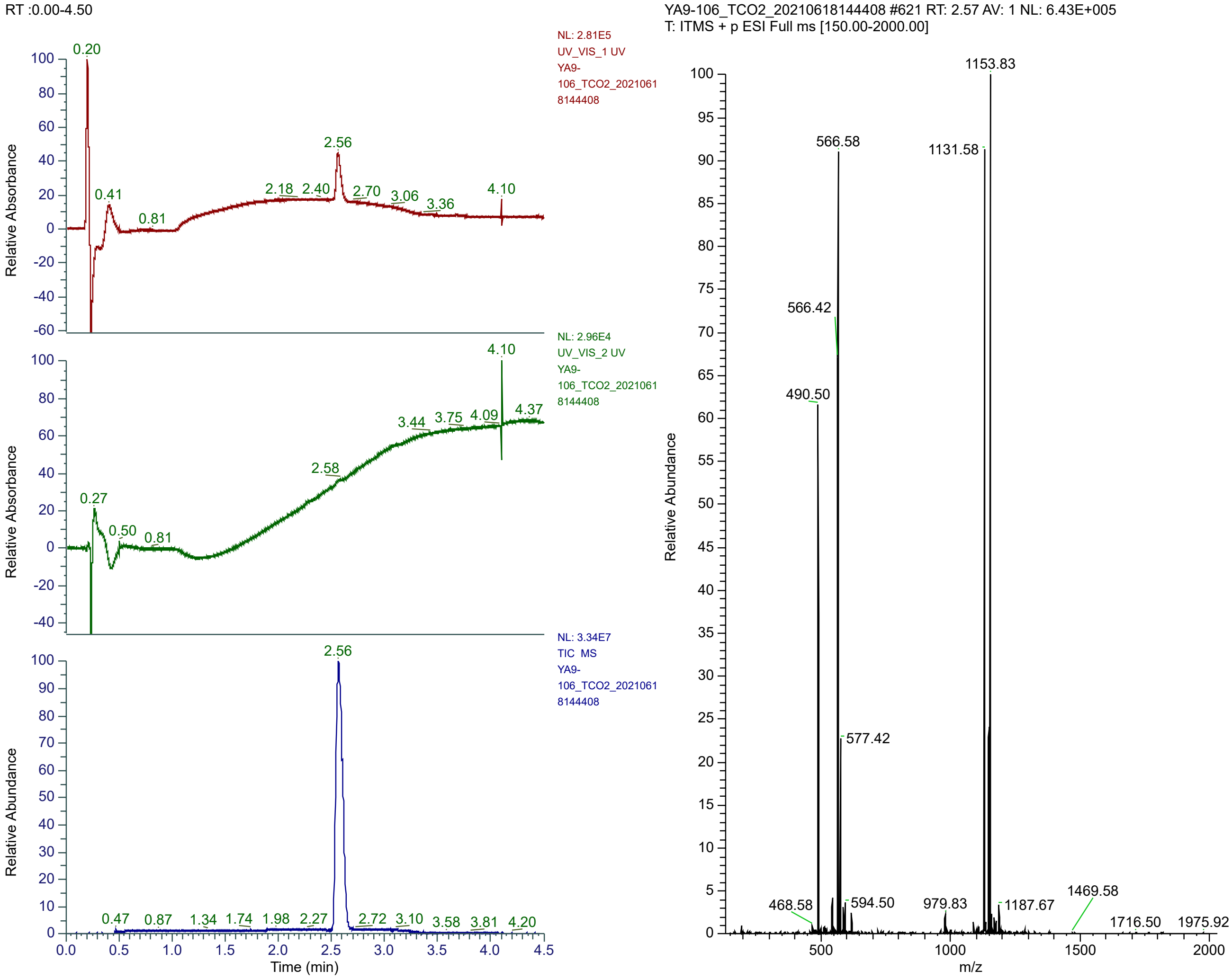

HPLC trace and low-resolution ESI-MS spectrum of pure product S11.

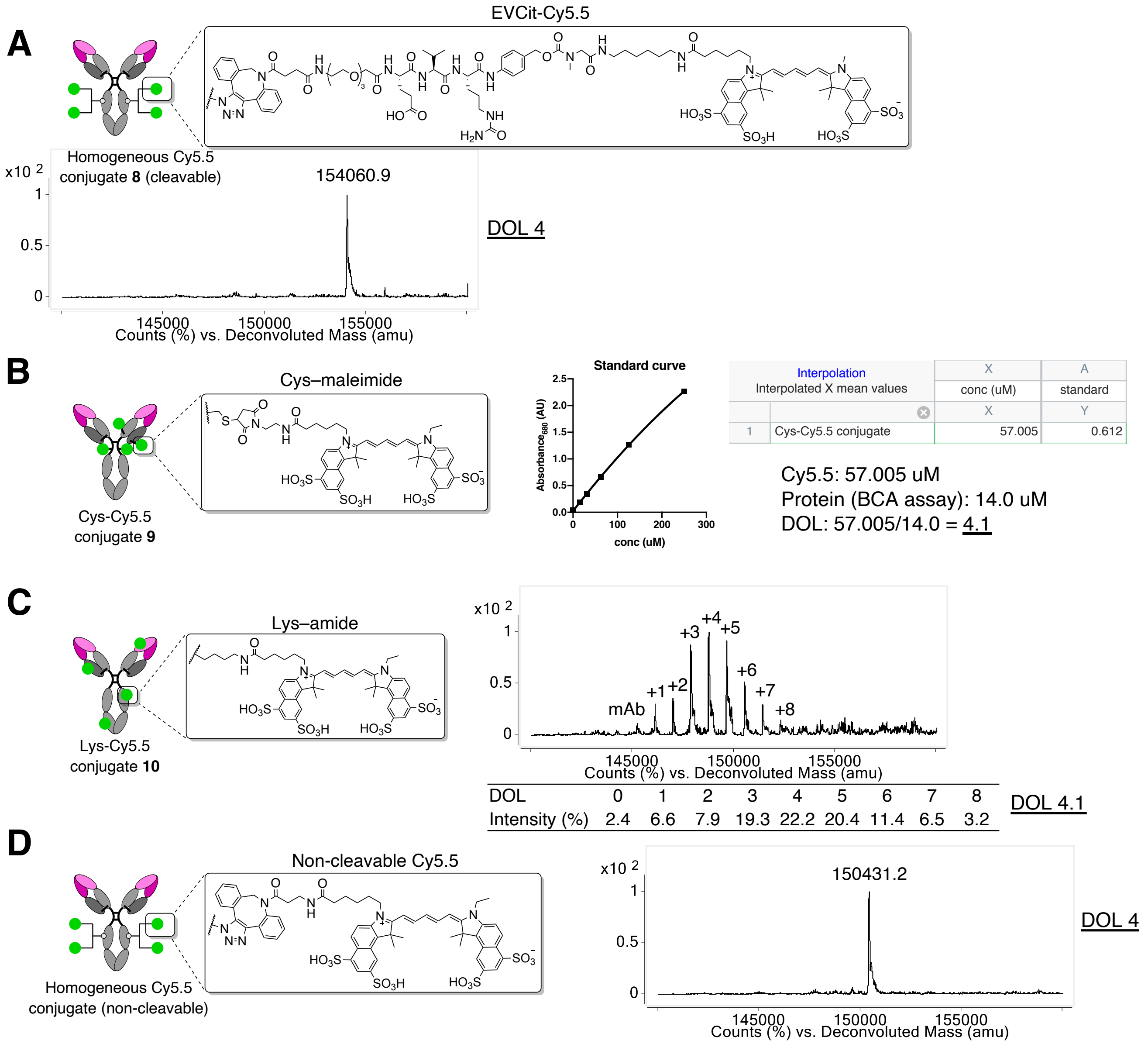

DOL	0	1	2	3	4	5	6	7	8
Intensity (%)	2.4	6.6	7.9	19.3	22.2	20.4	11.4	6.5	3.2

Characterization of Cy5.5 conjugates 8–10. (A) ESI-MS analysis of homogeneous cleavable Cy5.5 conjugate **8** with a DOL of 4. (B) Determination of DOL of Cys-Cy5.5 conjugate **9**. Protein concentration was determined by BCA assay and the molar amount of conjugated Cy5.5 was calculated using a free Cy5.5-based standard curve. The average DOL was then determined to be 4.1 based on the ratio of those values. (C) ESI-MS analysis of Lys-Cy5.5 conjugate **10**. The average DOL was determined to be 4.1 based on the ion intensity of each DOL species. (D) ESI-MS analysis of the homogeneous non-cleavable Cy5.5 conjugate with a DOL of 4.

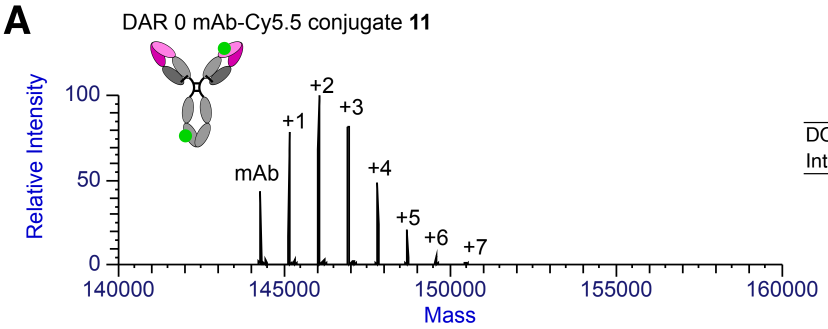

DOL	0	1	2	3	4	5	6	7
Intensity (%)	11.5	20.8	26.6	21.6	12.7	5.4	1.3	0.2

→ DOL 2.3

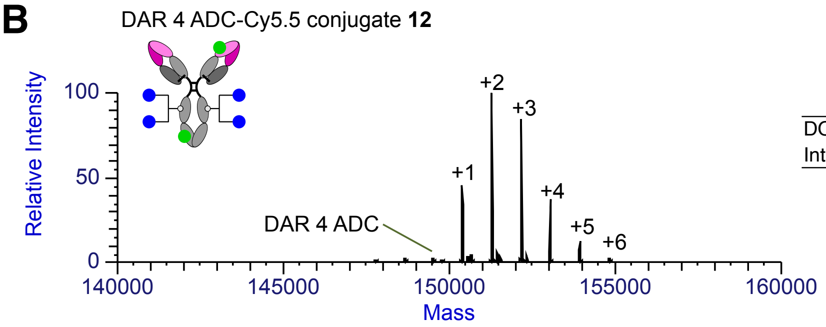

DOL	0	1	2	3	4	5	6
Intensity (%)	0.6	16.0	35.6	29.8	13.0	4.3	0.6

→ DOL 2.5

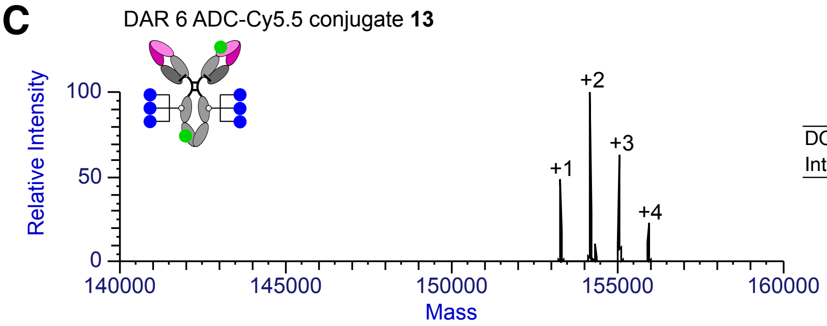

DOL	0	1	2	3	4
Intensity (%)	0	20.9	42.7	26.7	9.7

→ DOL 2.3

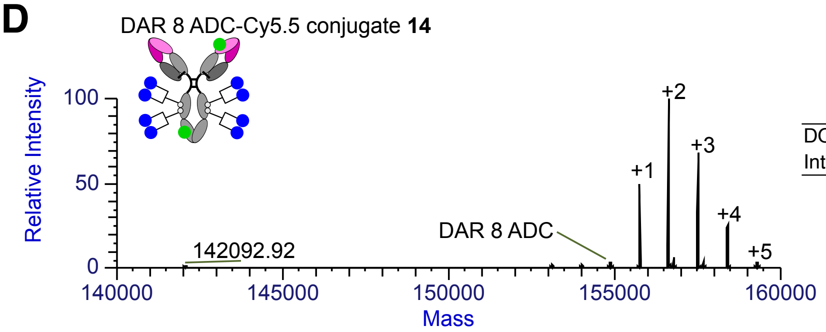

DOL	0	1	2	3	4	5
Intensity (%)	1.2	19.7	40.5	27.2	10.2	1.2

→ DOL 2.3

Characterization of Cy5.5-labeled MMAF conjugates 11–14. (A) ESI-MS spectra of anti-EGFRvIII mAb-Cy5.5 conjugate **11** with an average DOL of 2.3, (B) DAR 4 MMAF-Cy5.5 conjugate **12** with an average DOL of 2.5, (C) DAR 6 MMAF-Cy5.5 conjugate **13** with an average DOL of 2.3, and (D) DAR 8 MMAF-Cy5.5 conjugate **14** with an average DOL of 2.3.
